# Multi-Omics and Genome-Scale Modeling Reveal a Metabolic Shift During *C. elegans* Aging

**DOI:** 10.3389/fmolb.2019.00002

**Published:** 2019-02-06

**Authors:** Janna Hastings, Abraham Mains, Bhupinder Virk, Nicolas Rodriguez, Sharlene Murdoch, Juliette Pearce, Sven Bergmann, Nicolas Le Novère, Olivia Casanueva

**Affiliations:** ^1^Department of Epigenetics, Babraham Institute, Cambridge, United Kingdom; ^2^Department of Computational Biology, University of Lausanne, Lausanne, Switzerland

**Keywords:** metabolomics, flux balance analysis, aging, systems biology, multi-omics, whole genome model, *C. elegans*

## Abstract

In this contribution, we describe a multi-omics systems biology study of the metabolic changes that occur during aging in *Caenorhabditis elegans*. Sampling several time points from young adulthood until early old age, our study covers the full duration of aging and include transcriptomics, and targeted MS-based metabolomics. In order to focus on the metabolic changes due to age we used two strains that are metabolically close to wild-type, yet are conditionally non-reproductive. Using these data in combination with a whole-genome model of the metabolism of *C. elegans* and mathematical modeling, we predicted metabolic fluxes during early aging. We find that standard Flux Balance Analysis does not accurately predict *in vivo* measured fluxes nor age-related changes associated with the Citric Acid cycle. We present a novel Flux Balance Analysis method where we combined biomass production and targeted metabolomics information to generate an objective function that is more suitable for aging studies. We validated this approach with a detailed case study of the age-associated changes in the Citric Acid cycle. Our approach provides a comprehensive time-resolved multi-omics and modeling resource for studying the metabolic changes during normal aging in *C. elegans*.

## Introduction

The nematode *C. elegans* is widely used as a model organism to interrogate the molecular mechanisms of aging (Guarente and Kenyon, 2000). Many genes and interventions that prolong life, collectively called longevity pathways, are conserved across species, and cause global remodeling of metabolic pathways (López-Otín et al., 2016). To fully understand what makes a certain type of metabolism healthy, it is crucial to understand how it compares to metabolic changes that occur during the normal course of aging. Morphological changes evident in post-reproductive wild-type *C. elegans* point toward the loss of key metabolic capabilities during normal aging. For example, the intestine, which is the main metabolic organ in worms, undergoes atrophy with age (Ezcurra et al., 2018). Another key metabolic change is that mitochondria become fragmented and lose volume (Yasuda et al., 2006; Regmi et al., 2014) with a concurrent loss of electron chain oxygen consumption and ATP production (Braeckman et al., 2002; Houthoofd et al., 2005). Mitochondrial damage is also a hallmark of human aging (Chaudhari and Kipreos, 2018) and can be delayed using interventions that prolong life (Houthoofd et al., 2005; Brys et al., 2010). Various age-related pathologies can be observed in worms after the time of sperm depletion and are fully evident prior to median lifespan (Ezcurra et al., 2018). Consistently with these observations, functional assays have identified several metabolites that are able to delay aging in worms, e.g., oxaloacetate (Williams et al., 2009), alpha-ketoglutarate (Chin et al., 2014), and N-acylethanolamine (Lucanic et al., 2011).

Omics techniques can provide a global overview and aid in the elucidation of the causes underlying early decay. For example, metabolic changes that characterize wild type and long-lived *C. elegans* strains have been previously studied by metabolomics alone (Fuchs et al., 2010; Pontoizeau et al., 2014) as well as multi-omics approaches including metabolomics as one layer (Copes et al., 2015; Davies et al., 2015; Gao et al., 2017a,b; Wan et al., 2017). However, one common problem with omics applied to aging samples is that *C. elegans* is a hermaphroditic species and contamination of aging populations with progeny introduces a confounder. The use of 5′-fluoro-2′-deoxyuridine (FUDR) can circumvent this problem because it decreases progeny production by reducing germ cell division, however this treatment has been shown to directly influence metabolism (Davies et al., 2012; García-González et al., 2017; Scott et al., 2017). As a consequence, the early stages of the aging process are either under-sampled or have been subjected to a chemical intervention, clouding our view of the normal aging process. It is key to circumvent these technical problems because a better understanding of the mechanisms that explain early age-related decline can potentially lead to the development of interventions targeting the delay of aging and prevention of age-associated disease in humans.

Recent advances in modeling approaches enable the use of -omics datasets in conjunction with genome-scale metabolic models (GSMM) for *in silico* predictive studies. GSMMs are mathematical representations of all known metabolic reactions in an organism. Three such models have been recently published for *C. elegans* (Gebauer et al., 2016; Yilmaz and Walhout, 2016; Ma et al., 2017) and a community-driven approach has reconciled and extended these into a consensus model (Hastings et al., 2017; Witting et al., 2018). Whole-genome metabolic models can be used to predict intracellular turnover rates (fluxes) for metabolic reactions using for instance Flux Balance Analysis (FBA), a mathematical method that uses the stoichiometry of every reaction in a whole system to derive a steady state solution by optimizing an objective function, usually growth (Orth et al., 2010). To reduce the space of possible solutions, it is necessary to add constraints based on experimental data. Enzyme expression levels, provided by proteomics or transcriptomics data, are usually used as a proxy for the expected flux through any reaction catalyzed by that enzyme. Because FBA calculates the flow of metabolites throughout the global network, it allows the elucidation of how changes in one component of the model affect other pathways and phenotypes (such as growth rate or the rate of production of a certain metabolite), and in this way it can provide valuable mechanistic insights (Orth et al., 2010).

In this study we have used two infertile but normal-lived strains grown in the absence of FUDR, to conduct temporally resolved multi-omics studies, including transcriptomics, and targeted MS-based metabolomics. We characterize the changes over the course of aging in about 100 metabolites to find 44 that change significantly with age. Among these, more than half of them have already been characterized as longevity modulators. The data shows metabolic shifts during the course of aging, with a prominent decrease in the levels of aminoacids while their polyamine derivatives increase. In addition, metabolites involved in the TCA cycle become imbalanced before worms reach middle age. We harnessed this data using FBA during the course of normal aging before midlife. When looking at central carbon metabolism, we noticed that the model constrained by transcriptomics was unable to accurately match previous *in vivo* measured fluxes. While ordinarily, the objective function for FBA is linked to growth, for aging post-mitotic multicellular organisms, growth can no longer be assumed to be the appropriate objective. Thus, we create an objective function that qualitatively incorporates *in vivo* measured metabolomics information by instructing the model that if a metabolite level changes significantly between two time points, the associated reactions should either produce or consume that metabolite. We find that when using the TCA cycle as a case study, the usage of multi-omics data with FBA provides more accurate predictions than standard FBA, indicating that the new objective function is better suited for FBA studies in post-developmental worms. This optimized method correctly predicts that the most decreased fluxes within the TCA metabolic shift are through oxaloacetate, in line with experiments that show that it becomes the most limiting TCA-related metabolite in old animals. The model also provides a mechanistic explanation that links the decreased production of glutamate to a malfunctioning TCA cycle. In summary, we present a valuable multi-omics resource that can be used as a baseline for other early aging studies as well as an improved systems biology tool that provides a closer representation to the *in vivo* metabolism of normal lived animals.

## Materials and Methods

Additional methods can be found in Supplementary Material.

### Strains

List of strains used in this study:

GR1395 *mgIs49*[mlt-10p::GFP-pest; ttx-1p::GFP] IV
MOC001 GR1395 outcrossed to N2, 7 times
EJ1171 *gon-2(q388)*; *gem-1(bc364)*
JK816 *fem-3(q20)* IV
MOC91 *gon-2(q388); gem-1(bc364); mgIs49*[mlt-10p::GFP-pest; ttx-1p::GFP] IV

GR1395 was obtained from A. Frand's lab (Frand et al., 2005). The mlt-10p::GFP molting reporter was used to identify the exact molting times and ensure developmental timing did not differ between strains. EJ1171 was a kind gift from Eric Lambie's lab (Kemp et al., 2009); stocks were grown at 16°C in Mg^2+^ containing plates and thawed regularly. Before using them for experiments they were passed for 2 generations at 16°C in regular media without Mg^2+^. Conditional sterility was obtained by growing L1 larvae to adulthood at restrictive temperature (25°C). At 25°C the penetrance of gonadogenesis failure in *gon-2(q388)*; *gem-1(bc364)* is <100%, whereas for *fem-3(q20)* the penetrance is complete.

### Worm Maintenance and Sample Information

Worms were maintained at 16°C on NGM with OP50 *E. coli*. Synchronized experimental populations were prepared by washing gravid adults and eggs from plates and bleaching in a freshly prepared solution of 1% sodium hypochlorite and 1 M potassium hydroxide. Eggs were allowed to hatch overnight at 25°C in M9 solution (22 mM KH2P04; 42 mM Na2HP04; 86 mM NacL; 1 mM MgSo4) to ensure all animals arrested at the L1 stage. This produced a tightly synchronized population which was confirmed by mlt-10p::GFP fluorescence. Experimental populations were placed at 25°C on HT115 *E. coli* containing the empty vector plasmid L4440 on standard NGM plates containing 50 μg/ml Carbenicillin, 1 mM IPTG, and 10 μg/ml Nystatin until harvesting. For the *gon-2(q388); gem-1(bc364);* strain, samples were inspected before harvesting and visibly fertile worms were picked off. Plates with large numbers of visibly fertile worms were discarded. (Note that under high magnification a degenerate gonad could be seen in a subset of *gon-2(q388); gem-1(bc364);* animals at 117 h (D4) and were not discarded). Samples were harvested at the following hours post-feeding of arrested L1:

- Day 1: Hours 41 and 49 post feeding (3 replicates per strain per time point)
- Day 2: Hours 65 and 73 post feeding (3 replicates per strain per time point)
- Day 3: Hours 89 and 97 post feeding (3 replicates per strain per time point)
- Day 4: 117 h post feeding (3 replicates per strain)
- Day 5: 137 h post feeding (2 replicates per strain)
- Day 10: 257 h post feeding (2 replicates per strain).

### RNA-Sequencing

Samples of at least 1,000 worms were prepared as described in Supplementary Methods “RNA-Sequencing Library Preparation.” For PCR library enrichment, 13 cycles of amplification were performed. Library quality was assessed on a Bioanalyzer High Sensitivity DNA Chip (Agilent 5,067–4,626) and concentration was determined using KAPA Library Quantification Kit (KK4824). Libraries were sequenced on an Illumina HiSeq 2,500 system by the Babraham Sequencing Facility.

### RNA-Seq Data Analysis

RNA-Sequencing data was prepared and normalized as described in Supplementary Methods “RNA-Seq data preparation and normalization” and Figure S1. Briefly, raw reads were trimmed and mapped to the *C. elegans* WBCel235 genome assembly using HISAT2 (Liu et al., 2017). All libraries were mapped as non-directional single-end. Only exactly overlapping reads were assigned to a gene and transcript-isoforms were merged. Raw counts were generated using Seqmonk (Babraham Bioinformatics, http://www.bioinformatics.babraham.ac.uk/projects/seqmonk/) and all subsequent analysis was performed in R (http://www.R-project.org). The DESeq2 package (Love et al., 2014) was used to generate normalized counts.

### Metabolomics Assay

Targeted metabolomics using Mass Spectrometry was performed by the Northwestern Metabolomics Research Center (https://depts.washington.edu/mmcslu/resources/current-research/metabolomics/) from a batch containing ~2,000 worms per replicate. Worms were washed several times with M9 and snap frozen in water. Samples were homogenized with a bullet blender in 0.1x PBS at 4°C, protein precipitated with methanol, sonication, and centrifugation. The supernatant was removed and dried in a vacufuge (Speedvac) at 30°C. All samples were processed in parallel to avoid batch effects.

Targeted LC-MS/MS metabolomics targeting a list of 210 metabolites was performed on a system consisting of Shimadzu Nexera XR LC-20AD pumps coupled to a Sciex 6,500+ triple quadrupole spectrometer operating in MRM detection mode through the Sciex Analyst 1.6.3 software. The system includes a dual column setup with dedicated columns for positive ionization mode and negative ionization mode. The results for each sample are therefore the result of two injections. Metabolite concentrations were quantified using Multiquant 3.0 software in relative manner. The samples were separated on a Waters Xbridge BEH amide column (2.5 um, 130 angstroms, 2.1 x 150 mm) operated in a HILIC regime at 40 C. Solvent A consisted of 95% water, 3% acetonitrile, 2% methanol, 0.2% Acetic Acid (v/v/v/v) 10 mM ammonium acetate, pH ~4.2. Solvent B consisted of 93% acetonitrile, 5% water, 2% methanol, 0.2% acetic acid, and 10 mM ammonium acetate. Organic solvents and acetic acid were Optima grade from Fisher Scientific USA, ammonium acetate was from Sigma Aldrich. 18.2 MOhm water was from a Synergy UV system by Millipore. Gradient at 0.300 mL/min was as follows: 0–3 min 95% B, 3–8 min 95–50% B, 8–12 min 50% B, 12–13 min 50–95% B 13–18.1 min 95% B. During the injection on columns of opposite polarity solvent continued at 95% B giving each column ~23 min of equilibration time. Samples were normalized to total protein content quantified by Bradford assay.

### Metabolomics Data Analysis

Metabolomics data analysis was performed using R (http://www.R-project.org). As is typical in this type of metabolomics data, there were several missing values in the dataset. We removed metabolites that had more than 10% such missing values across all samples, leaving 105 metabolites. For the remainder of the missing values, we interpolated them by replacing missing values with the row (metabolite) mean (across all samples) so as not to affect downstream analyses. This is a standard practice in metabolomics analyses. The dataset was then transformed using the inverse hyperbolic sine, which is linear for small *x* while asymptotically approaching log(2*x*). In particular, zero values are mapped to zero and all other values are mapped to positive values (which is not true for log-transformation). The data were further normalized by mean-centering and scaling so that every metabolite had a mean of 0 and a standard deviation of 1, rendering the values comparable. The normalized dataset can be found in Supplementary Table 4. Initial investigation of the dataset indicated that one sample was an outlier needing to be removed as it was separated from the remainder of the dataset in the unsupervised PCA, and moreover had a high within-sample coefficient of variation.

### Analysis of Variability Drivers–PCA, Distances, Correlations

Principal component analysis (PCA) was conducted for both the transcriptomics and the metabolomics datasets using the “prcomp” package in R and the samples were visualized as distributed in the first two principal components. Sample to sample correlations were calculated and plotted using the “corrplot” package in R. Sample to sample distances were calculated using 1-cor(*x*,*y*) for each pair of samples *x* and *y*. The density plot of sample to sample distances was plotted by categorizing the pairs (*x*,*y*) of samples according to whether they were replicates or had the same age (but different strains) or had the same strain (but different ages).

### Data Availability

The RNA-Sequencing dataset is available from the Gene Expression Omnibus (GEO) database with accession GSE124994 (http://www.ncbi.nlm.nih.gov/geo/query/acc.cgiacc=GSE124994). The metabolomics dataset is available as a Supplementary File (see Supplementary Materials for details).

### Determination of Age-Associated Metabolites

To determine which metabolites were significantly changing with age in our metabolomics dataset, partial least squares discriminant analysis (PLS-DA), as implemented in the R package “ropls” (Thévenot et al., 2015), was used with normalized metabolite abundances, with the sample time in hours as the variable. PLS-DA is a latent variable regression method based on covariance between the predictors and the response, has been shown to efficiently handle datasets with multi-collinear predictors, as in the case of spectrometry measurements (Wold et al., 2001). PLS-DA is a linear regression-based method, thus non-linear effects with respect to aging will be overlooked. The Variable Importance in Projection (VIP), reflects both the loading weights for each component and the variability of the response explained by this component and can be used for feature selection (Pinto et al., 2012). The heat map of age-associated metabolites was plotted using the R function “heatmap.2.”

### Flux Balance Analysis (FBA) Pipeline

We used the WormJam *C. elegans* whole-genome metabolic reconstruction, version dated 25-01-2018. This model includes 3,301 reactions and 2,393 metabolites (1,334 unique metabolites after discarding duplication of metabolites across compartments). The CobraPy library in Python (Ebrahim et al., 2013) was used to run FBA and as a basis for the implementation of FBA-associated methods, including integration of transcriptomics and metabolomics data. All sources of input (uptake) were constrained to zero except oxygen (maximum of 100 units allowed uptake), bacterial input as food source (maximum of 10 units allowed uptake) and trace minerals and ions (Ca^2+^, Cu^2+^, K^+^, Mg^2+^, Mn^2+^, Na^+^, Zn^2+^; arbitrary allowed uptake). The biomass reaction used as the default objective function included glycans, phospholipids, collagens, DNA, RNA, free fatty acids, glycogens, proteins, triacylglycerols, and trehalose. FBA was executed using COBRApy's parsimonious FBA method “cobra.flux_analysis.pfba.”

### Integration of Transcriptomics Data With FBA

The transcriptomics data were first log-transformed and then means were obtained for biological replicates in order to have a single expression value per time point and strain. These were integrated with the model using a two-step approach. First, a context-specific model was built by removing reactions mediated by genes whose expression was below a threshold (Akesson et al., 2004). Then for the remaining reactions, the E-flux approach (Colijn et al., 2009) was used, in which reaction upper (and lower if the reaction is reversible) bounds are set in proportion to the expression level. Where multiple genes were annotated to a reaction using OR-logic (i.e., any of the genes may catalyze the same reaction), their expression levels were added together to obtain the reaction constraint. For complexes, where multiple genes are annotated to the reaction using AND-logic (i.e., all of the genes are needed to catalyze the reaction), the minimum of their expression levels was used to obtain the reaction constraint. In total, 2,178 reactions in the model were constrained by transcriptomics information. The bulk of the remaining reactions had no annotated genes; only seven reactions had genes annotated that were absent in the transcriptomics dataset.

### Integration of Metabolomics Data With FBA

There is no direct correspondence between metabolite levels and fluxes in FBA. Previous approaches to integrate metabolomics data with FBA include: (1) requiring that detected metabolites are produced in non-zero quantities by the model (e.g., GIM3E, Schmidt et al., 2013), (2) calculating a rate of change for a metabolite from a time series or between two time points, and requiring the production of the metabolite by the model to correspond to the rate of change [e.g., TREM-flux, Kleessen et al., 2015, and (3) “unsteady state” FBA, Bordbar et al., 2017]. Our approach is similar to those mentioned in (2) in that we require a time series of metabolomics measurements, but in contrast to those approaches we do not use the metabolomics values to *constrain* the model, but rather to alter the *objective*. We chose this approach because our first experiments using FBA on this dataset showed that using a constraints-based metabolomics data integration approach would lead to the model not being able to grow at some of our time points, as incompatible constraints are generated (Figure S9B). While ordinarily, the objective function for FBA is linked to growth, for multicellular organisms, growth can no longer be assumed to be the sole cellular objective and other phenotypically appropriate proxies are needed. We consider that, from a cellular perspective, if a metabolite level has increased between two time points, then this implies that within that timeframe the sum of fluxes through all reactions producing that metabolite (i.e., its supply) must have exceeded the sum of the fluxes through all reactions consuming it (i.e., its demand). Conversely, if the metabolite level decreased, then within that timeframe, the demand must have exceeded the supply (further described in the text and in **Figure 6**). Accordingly, we added the net-production or net-consumption of changed metabolites as an *additional* objective function alongside the production of biomass, simultaneously maximizing biomass and production or consumption of the relevant metabolites.

Our method can be formulated mathematically as follows:

∀i ∈(2 ≤i ≤N),

$$maximise\;\left(c^{T}v\;+\;u^{T}v-d^{T}v\;\right)$$

s.t.

Sv=0 ,

$$tr^{L}\leq v\;\leq tr^{U}$$

$$\;u^{T}=\;\left\{\forall m\in M,\;1\;if\;x^{m_{i}}>x^{m_{i-1}}\;,else\;0\right\},$$

$$d^{T}=\left\{\forall m\in M,1\;if\;x^{m_{i}}<x^{m_{i-1}},\;else\;0\right\}$$

where *N* is the total number of time points, *i* is the index of a particular time point, c^T^ is the vector of coefficients of the biomass equation, u^T^ is the vector of coefficients for demand reactions corresponding to the metabolites with level increases between time points *i* and *i-1*, d^T^ is the vector of coefficients for demand reactions corresponding to the metabolites with level decreases between time points *i* and *i-1*, S is the stoichiometric matrix for all reactions in the model, *v* is the vector of fluxes, *M* is the set of all measured metabolites that could be mapped to the model, ${\text{x}}^{{\text{m}}_{\text{i}}}$ is a metabolite level for a particular metabolite *m* at a given time point *i*, and tr^L^, and tr^U^ are flux lower and upper constraints set from the transcriptomics data using the method described in the previous section.

For each metabolite, a *t*-test was used to determine which metabolite levels had changed significantly between consecutive time points. For two of the total number of time points per strain, we had only two replicates, insufficient for a statistical test of this type, thus the strains were combined to give at least four data points per time point. This was justified by our finding that strain differences are not the main driver of variability in the measured metabolome. A comparison of the means was then used to determine if the metabolites with level changes had increased or decreased. We were only able to do this for (*N*-1) of the *N* time points; flux predictions at the initial time point are thus unaffected by the metabolomics data. Moreover, there were no significant metabolite level changes between time points 89 and 97 h (day 3). Only metabolites which could be mapped to model metabolites were included: this was the case for 75 of the 105 measured metabolites. Of those, 63 metabolites had at least one significant between-time-point difference, while 12 were unchanged across all time points.

## Results

### Standard FBA Does Not Accurately Recapitulate Fluxes Measured *in vivo* in Adult Animals

**Figure 1:**
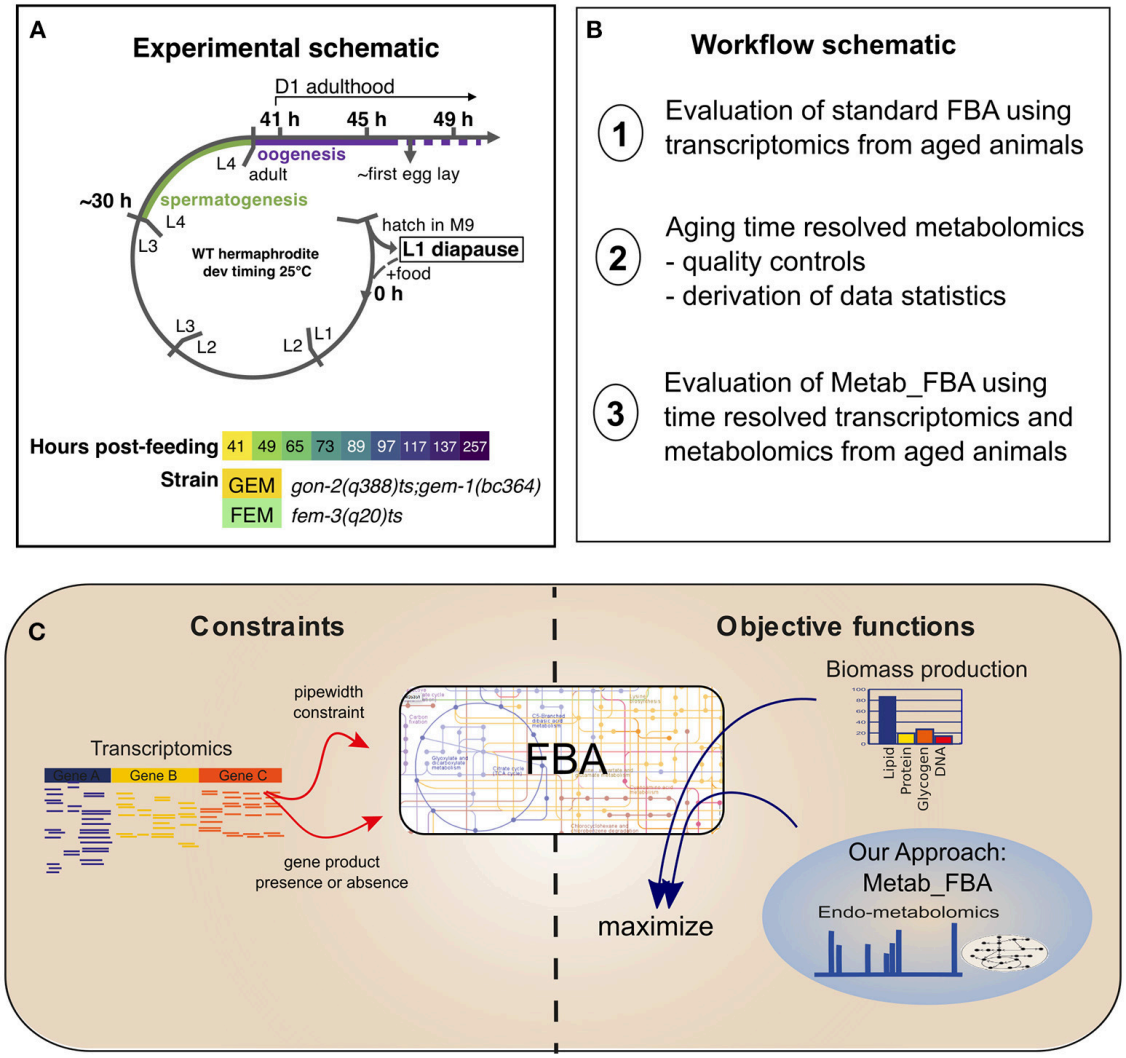
Experimental schematic. **(A)** Our experimental design included two sterile strains with differing gonadal composition sampled from early D1 of adulthood (41 h post-L1-feeding, which coincides with the very end of the L4 to adult molt) to D10 of adulthood (257 h post-L1-feeding). For days 1 through 4, triplicate samples were obtained. For days 5 and 10, duplicate samples were obtained. L1–L4 correspond to larval stages 1–4, L1 diapause is induced by hatching eggs in the absence of food. **(B)** Schematic of the workflow of this article. **(C)** Diagram representing the methodologies used for FBA. Standard FBA uses only biomass production as an objective function, while our approach combines the standard objective function with metabolomics data.

In order to study the dynamics of metabolic remodeling during aging on normal lived animals, we generated linked, temporally resolved, transcriptomic, and metabolomic data from two normal lived sterile strains, covering days 1–10 of adulthood (Figure 1A). *C. elegans* is a hermaphrodite species that produces progeny through self-fertilization, which can contaminate the aging parental samples. To avoid this confounding factor, we used two conditionally sterile strains, *gon-2(q388)ts; gem-1(bc364)* (GEM, hereafter), and *fem-3(q20)ts* (FEM, hereafter). The *gon-2* gene encodes a cation channel required for division of postembryonic gonadal precursor cells (Sun and Lambie, 1997). At restrictive temperature, the *gon-2(q388)ts* mutation is enhanced by mutation of the solute carrier *gem-1* (Kemp et al., 2009) but remains incompletely penetrant, resulting in delayed and degenerate gonadogenesis. Thus, GEM animals possess a degenerate gonad that by D4 might contain both sperm and oocytes, and, in a small number of cases, embryos. The *fem-3* gene encodes a novel sex-determination gene and the gain-of-function *fem-3(q20)ts* mutation causes temperature sensitive masculinization of the germline (Barton et al., 1987). Thus, FEM animals possess a normal hermaphrodite somatic gonad that produces no oocytes but excess sperm and are 100% sterile. Survival analysis confirmed that these strains display WT lifespan (Figure S2). During sample collection, each biological replicate was partitioned so that both metabolomics and transcriptomics data were produced from the same batch of worms.

Transcriptomics data can be used in combination with whole genome models for the computational prediction of fluxes (intracellular turnover rates) for metabolic reactions using FBA and this approach has been previously used to predict fluxes in normal and perturbed aging in worms (Gebauer et al., 2016). Two assumptions are key to FBA. The first is that the modeled system has reached a steady state (i.e., metabolite levels are no longer changing), and the second is that the system has been optimized through evolution to achieve some goal, usually considered to be biomass production. The second assumption can be called into question for aging animals, because they often live well beyond the cessation of both growth and reproduction. For FBA to be useful, it has to provide mechanistic insights into metabolism and the accuracy of the predictions can be benchmarked against many features including gene essentiality (Opdam et al., 2017) and recapitulation of metabolic states when a certain reaction has been removed (Gebauer et al., 2016).

We performed standard FBA (Supplementary Table 1) using transcriptomics data obtained from FEM animals using the whole-genome model of *C. elegans* metabolism, as described in the Methods. As objective function, we maximized biomass production (growth). We first performed flux-based pathway analysis to detect pathways that were significantly changed in day 10 compared to day 5 FEM animals (Figure S3C and Supplementary Table 2). We found that the most enriched include fluxes through pathways that are expected to change with age such as purine and pyrimidine and amino acid metabolism, oxidative phosphorylation, glutathione metabolism, and the TCA cycle. Similar predictions were reached in previous studies where FBA was applied to aging networks (Gebauer et al., 2016), indicating that standard FBA can recapitulate overall metabolic functionality.

However, the best method to ascertain FBA performance is to compare predicted fluxes with *in vivo* measured fluxes. Here, we took advantage of a previous study where mass spectrometry was used to quantify fluxes using the relationship between a labeled precursor (glucose, provided in the food) and several products of the TCA cycle (Schrier Vergano et al., 2014). The TCA cycle is composed of a series of enzymatic reactions that include the core of aerobic respiration in the mitochondria (a diagram in Figure S3A). The previous metabolic flux profiling directly measured fluxes through the TCA cycle in wild type worms as well as in mutants for *isocitrate dehydrogenase* (*idh-1)*, the enzyme that produces alpha-ketoglutarate from isocitrate (Figure S3A, reaction 3) (Schrier Vergano et al., 2014). In the absence of this upstream enzyme, significant changes in fluxes were identified that led to the accumulation of lactate, fumarate, succinate, malate and in the depletion of glutamate, and aspartate. We reasoned that if FBA was properly recapitulating fluxes, then an *in-silico* removal of *idh-1* enzyme should render fluxes that are similar to those directly measured *in vivo*. As a first approximation, we thus determined if standard FBA could recapitulate the fluxes observed *in vivo* in *idh-1* KO animals, when performing *in silico* knock out of *idh-1* and *idh-2* in young adult FEM animals. We observed that, as expected, the loss of IDH activity eliminated fluxes through the reaction it catalyzes (making alpha-ketoglutarate from isocitrate) (Supplementary Table 3 and Figure S3B). In addition, *in silico* lack of alpha-ketoglutarate concurred to some extent with *in vivo* metabolic flux experiments, causing a reduction in the fluxes that lead to glutamate production, and an increase in citrate production. However, many of the altered fluxes measured *in vivo* (Figure S3A), including those leading to accumulation of succinate and malate, were not accurately reproduced by the *in silico* knock out (Figure S3B).

Because *in vivo* fluxes were measured in wild type (N2) young adult animals lacking *isocitrate dehydrogenase* activity, and the *in silico* experiment was performed using a transcriptome obtained from FEM young adult animals, one possibility is that the unmatched genetic backgrounds explain the differential fluxes. We think this is unlikely to be the main reason for the discrepancy, because the Pearson correlation of gene expression values between wild type and FEM animals at day 1 of adulthood is close to 98%, and samples only begin to diverge more significantly after day 2 of adulthood (data not shown). Although it remains a possibility, this *in silico* experiment suggests that although standard FBA can correctly predict global fluxes in aging animals, when looking at the results at a finer scale, the predictions may not be accurate. One likely scenario is that an objective function geared only toward biomass production may not appropriately reflect the biology of post-mitotic somatic cells in adult aging animals, and that new objective functions are required to study metabolism during aging. To obtain a more accurate objective function, we enhanced our FBA pipeline by integrating a metabolomics dataset into the objective function together with the biomass production (Figures 1B,C). As described below in the “Case study” section, the new objective function was further validated entirely within the FEM genetic background.

### Different Sources of Variance for Metabolome and Transcriptome in Linked Samples

**Figure 2:**
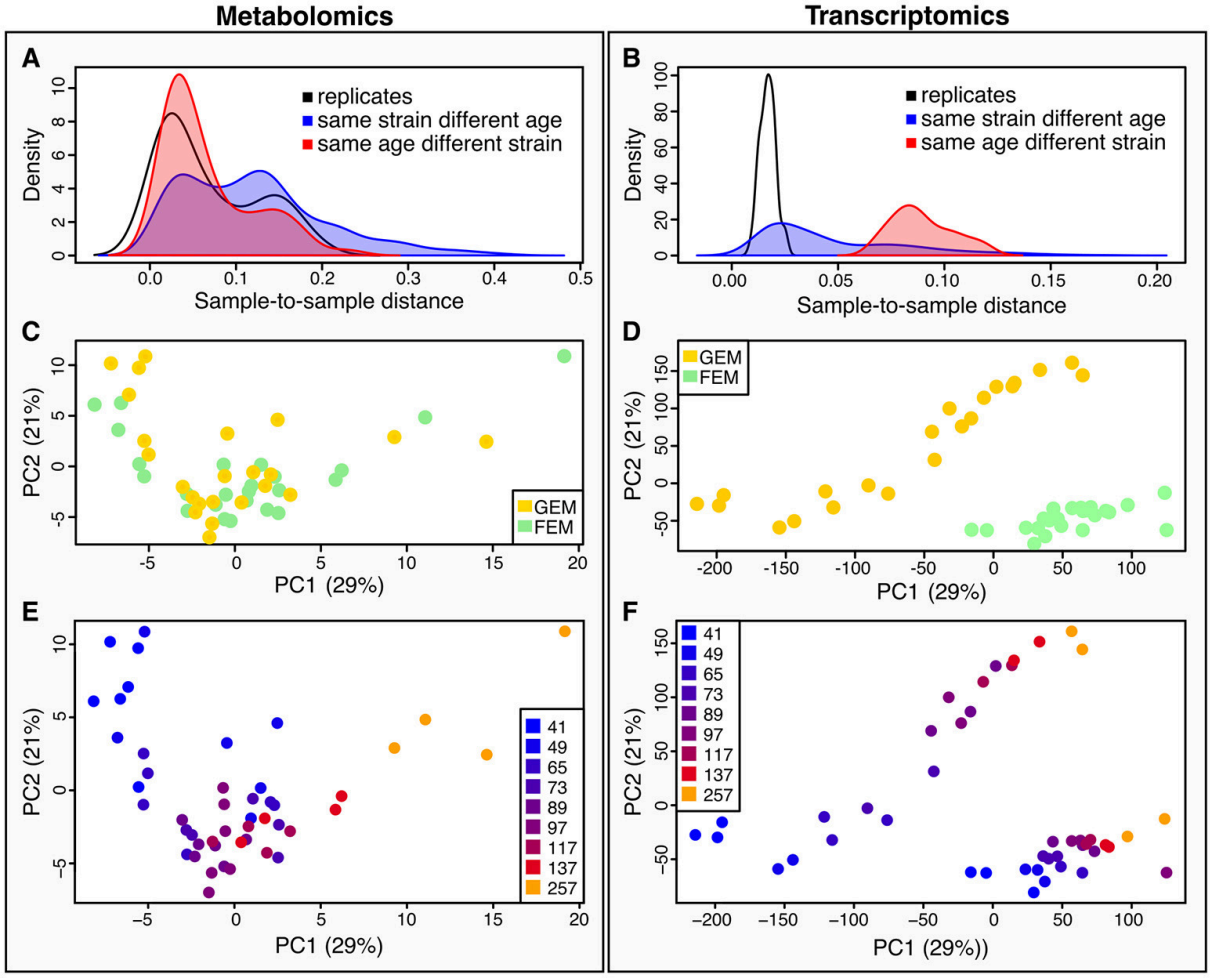
Characteristics of between-sample variability compared between metabolomics and transcriptomics datasets. **(A,B)** metabolomics **(A)**, and transcriptomics **(B)**, show density plots of the distributions of pairwise sample-to-sample distances as grouped by samples that were replicates (black line), or shared their age (but not strain) (red line and shaded area), or shared their strain (but not age) (blue line and shaded area). The density distribution being shifted toward the left in these plots means that the samples were more similar (had a lower distance). These plots show that for the metabolomics dataset **(A)**, samples with the same age but different strains were closer than those with the same strain but different ages, while the opposite is true for the transcriptomics **(B)**. This plot also illustrates that there is greater technical variance in the metabolomics dataset than for the transcriptomics. **(C,D)** we conducted Principal Components Analysis (PCA) across the two datasets [**(C)**, metabolomics; **(D)** transcriptomics]. We visualized the samples projected onto the space of the first two principal components. Samples are colored by strain–yellow for GEM, green for FEM. **(E,F)** the same principal components as in **(C)** and **(D)** are shown colored by age. [**(E)** metabolomics; **(F)** transcriptomics].

Quantification of metabolites is key for our understanding of metabolism during aging. Metabolites are the downstream product of the combined effect of transcriptional, post-transcriptional, and post-translational events, together with the influence of the environment, and therefore give a more immediate picture of the metabolic and physiological state, that may or may not correlate with corresponding transcripts. To determine how metabolomics data compares to transcriptomics, we calculated sample-to-sample distances for both datasets (Figure 2). As shown by the density plots, variability is affected by age and by genotype. However, the effect of age was greater than the effect of strain in the metabolomics dataset (Figure 2A) whereas, variance was more striking by genotype rather than age in the transcriptomics dataset (Figure 2B).

When using Principal Components Analysis (PCA; Figures 2C–F) to look at the main determinants of variability in the dataset, it is apparent that in the metabolomics data (Figure 2E), the aging process is the major contributor to variability between the samples, since the first principal component, accounting for the majority of variability largely aligns with age. On the other hand, the transcriptomics displays a large division across both PC1 and PC2 by strain, although age still accounts for a large proportion of the variability (Figure 2F). This difference in drivers for the between-sample variability cannot be due to batch differences in the underlying sample material because the transcriptomic and metabolomic samples were linked. We explore potential explanations for these observations in the Discussion section. Figure S4 shows the sample-to-sample correlation matrices for all samples in the metabolomics (Figure S4A) and transcriptomics (Figure S4B) datasets.

### Age-Associated Metabolites Are Enriched for Those Known to Promote Longevity

**Figure 3:**
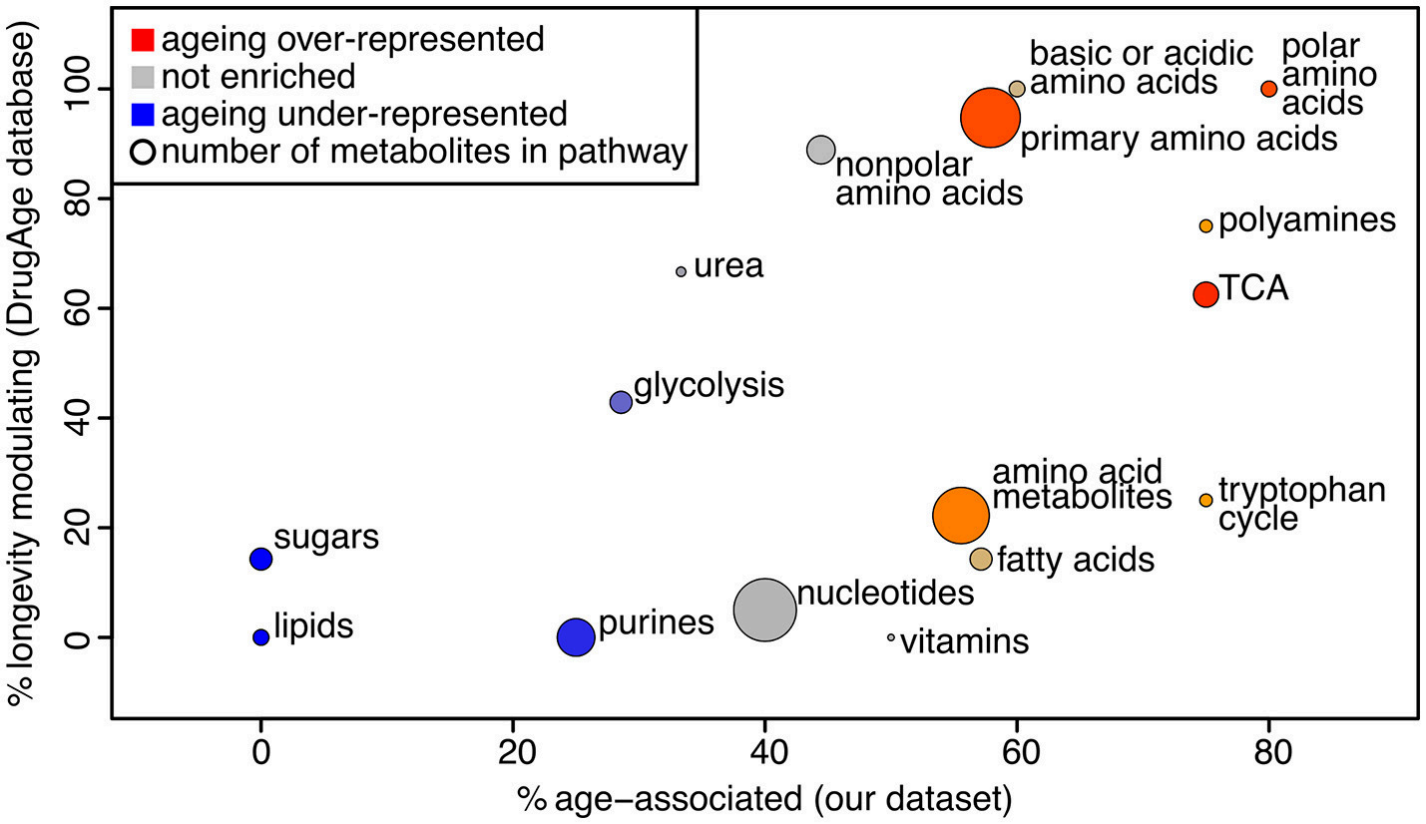
Bubble plot of pathway enrichment for age-associated metabolites. The pathways that are most red are the ones that have the most age-associated metabolites, while the ones that are the most blue have the least. Circle radius represents the number of measured metabolites in the pathway. The x axis shows the proportion of metabolites in the pathway that are changing intensity with age in our study, and the y axis shows the proportion of metabolites in the pathway that are known to modulate longevity in the Drug Age database, illustrating that these measures are broadly correlated.

We used PLS-DA to reveal the metabolites that are significantly associated with the age of the sample (statistical details in Supplementary Methods, Figure S5). This gave 44 metabolites presenting a significant change over the course of aging (Supplementary Table 5). Note that PLS-DA is a linear regression-based method, thus non-linear effects with respect to age will be overlooked. To determine whether those metabolites are likely to influence the aging process, we determined if these were present in a database for metabolites known to influence longevity (longevity modulators, determined from DrugAge as described in the Methods). Twenty of our 44 age-associated metabolites were known for their effect on longevity, more than would be expected by chance since only 34 of all 105 measured metabolites were in the database (Fisher's exact test for over-representation gives *p* = 0.013). Using a comprehensive search of the literature we found 6 additional metabolites amongst our age-associated metabolites that act as longevity modulators when supplemented (Supplementary Table 5). To determine which pathways were most enriched for age-associated metabolites, we performed pathway enrichment analysis (Figure 3). The results show that there is a correlation between metabolites that are age-associated in our dataset and longevity modulators in the DrugAge dataset.

**Figure 4:**
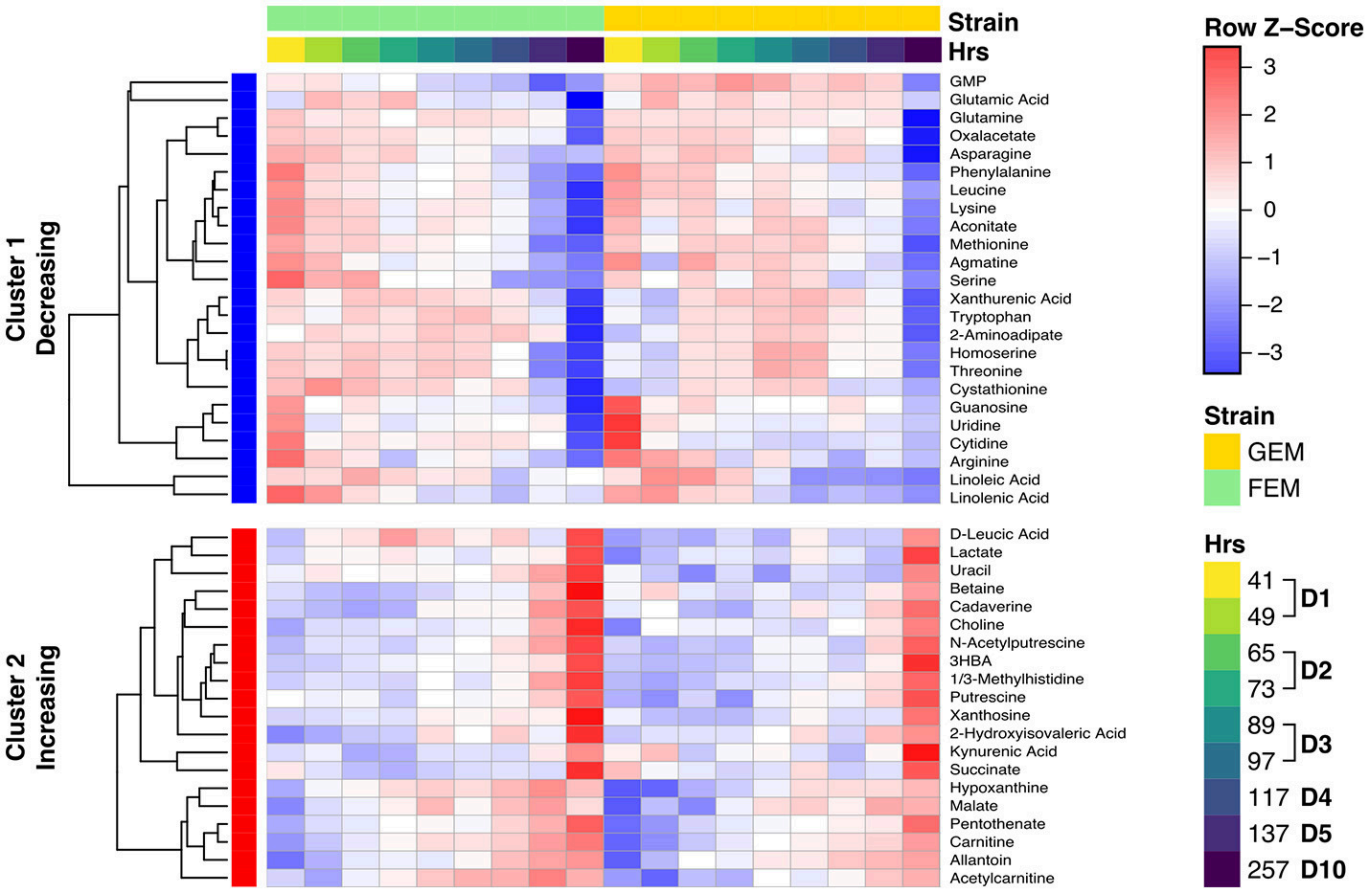
Clustered heat map showing metabolite levels over time. The heat map shows row (metabolite) Z-scores, i.e., deviations from the average across time points for each metabolite. Each metabolite range is scaled so that it has mean of zero and standard deviation of one. Each time point represents the average for biological replicates. The samples are ordered first by strain (green is FEM, yellow is GEM), then by age. The row sidebar illustrates whether the metabolites have been determined to be increasing or decreasing with age (red for increasing, blue for decreasing).

The age-associated metabolites cluster into two main groups: those that increase with age, and those that decrease with age, as shown in the clustered heatmap (Figure 4). Overall the data indicates that there is a clear shift for many metabolites. Two patterns are readily evident. Some metabolites drift with age (a gradually change for linoleic acid, hypoxanthine, and malate) whereas, others show abrupt shifts at specific time points during early or late aging. For example, the levels of several amino acids drop at day 1 of aging (between 41 and 49 h), and then again sometime after day 5 (137 h).

Our study generally agrees with other studies (Copes et al., 2015; Gao et al., 2017a; Wan et al., 2017) and a cross-comparison can be found in in Supplementary Table 6. The first cluster (cluster 1 in Figure 4) of metabolites decreasing over time contains most measured amino acids (including serine, threonine, leucine, lysine, glutamate/glutamic acid, methionine, tryptophan, and arginine) and some byproducts of amino acid metabolism (homoserine, cystathionine) as well as nucleotides (guanosine, cytidine, uridine, GMP). The second cluster (cluster 2 in Figure 4) of metabolites increasing with time contains degradation products of both nucleotides (hypoxanthine, xanthosine, and allantoin) and amino acid metabolism (betaine, carnitine, leucic acid, pentothenate, kynurenic acid, and xanthurenic acid), confirming a well-documented imbalance in amino acid and nucleotide metabolism with age (Copes et al., 2015; Gao et al., 2017a; Wan et al., 2017). The age-related change in amino acids has been proposed to be driven by the ratio of hydrophilic/hydrophobic surfaces that accompanies cell volume changes related to growth, and not by either age (Copes et al., 2015) or genotype (Gao et al., 2017a). As shown in Figure S6, there is a significant change in the worm's size from days 1–5, but body size remains stable thereafter. Therefore, when comparing days 5 and 10, something other than changes in cell size must underlie amino acid depletion in old animals. The large drop in these metabolites may cause them to become metabolically limiting as previous studies have shown that supplementation of specific amino acids and nucleotides can extend lifespan (Copes et al., 2015; Edwards et al., 2015).

We report here, for the first time, that there is accumulation of polyamines with age, namely, putrescine, N-acetylputrescine, and cadaverine. These metabolites display a sharp increase at day 10 (Figure 4) while an intermediary, agmatine, decreases significantly with age. Polyamines are low molecular weight aliphatic polycations, derived from amino acids, ubiquitous across species, and with many cellular functions. Putrescine is metabolized from arginine via either agmatine or ornithine and gives rise to spermidine, the precursor of spermine (Figure S7A), while cadaverine is the product of lysine decarboxylation (McCann, 1982). To check whether the observed metabolite changes aligned with changes in the corresponding metabolic gene pathways, we integrated the two datasets using the R library Pathview. As shown in Figure S7A, displaying the overlay of transcriptomics and metabolomics using Pathview, there is a significant age-dependent upregulation in the levels of spermidine synthase (see EC 2.5.1.16 in Figure S7A) in day 10 animals. This enzyme catalyzes the transfer of a polyamine group from S-adenosylmethioninamine to putrescine in the biosynthesis of spermidine, possibly reflecting an increased usage of spermidine with age. The polyamine spermidine has been associated with lifespan extension by activating autophagy across species (Eisenberg et al., 2009; Minois, 2014) and the implications of an increase in polyamines with age are further explored in the Discussion section.

As shown in Figure 4, many metabolites that are involved in central carbon metabolism, particularly the TCA cycle, were significantly changed with age, e.g., lactate and oxaloacetate. An imbalance in the TCA cycle in 10-day old animals has been previously described (Wan et al., 2017) and we observed in our metabolomics data a consistent change in both FEM and GEM with age, indicating that changes were independent of the genotype. Overall, there was a striking change in TCA metabolites in 10-day adults compared to day 5 or younger worms and some of the changes observed were in line with changes in *idh-1* mutants (Figure S8). These metabolic changes may reflect the widespread fragmentation of mitochondria that begin at around this stage (Yasuda et al., 2006; Regmi et al., 2014). Pathview analysis indicated that most detected transcripts for TCA enzymes show decreased levels with age (Figure S7B) with the exception of citrate synthase, which catalyzes the first step of the TCA cycle condensing acetyl-CoA and oxaloacetate to form citrate. This suggests that citrate accumulates with age, although we did not directly measure it. Pathview analysis also revealed that for succinate there was a mismatch between the transcripts of the enzymes required for succinate production and consumption, and the levels of the metabolite, perhaps reflecting post-transcriptional alterations to the key enzymes.

### Flux Balance Analysis Performed With Metabolomics-Integrated Objective Function Reveals Dynamic Metabolic Flux Changes During Aging

**Figure 5:**
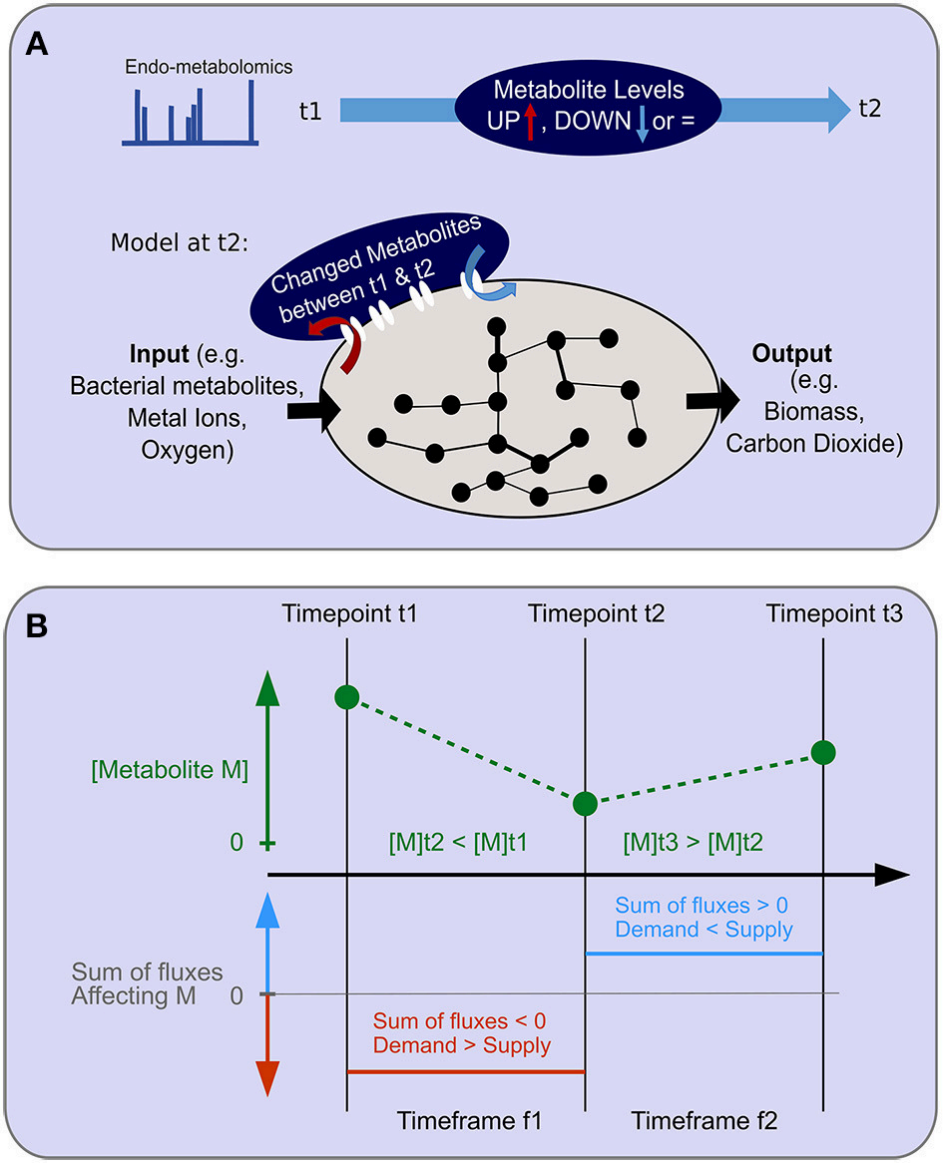
Objective function for endo-metabolomics inclusion. **(A)** A schematic of the objective function for endo-metabolomics inclusion into FBA is shown. Endo-metabolomics metabolite levels are obtained for each time point. Between each pair of consecutive time points (illustrated as t1 and t2), a statistical test reveals for each metabolite whether the level has increased (UP) or decreased (DOWN) between those two points. The integration of this information with the model uses the transcriptomics constraints from the second time point (t2) as an imation for the time frame (t1–t2) and adds a new system boundary as an additional objective function to reflect the changes in metabolite levels either as inputs or outputs to the system. **(B)** The relationship between metabolite level changes between consecutive time points and the assumed flux over the time frame is illustrated. An example of two consecutive level changes are shown for the hypothetical metabolite M which has a level decrease between time points t1 and t2, and a level increase between time points t2 and t3. During the time frame f1 for the duration between time points t1 and t2, demand exceeds supply. During the time frame f2 for the duration between time points t2 and t3, supply exceeds demands.

Standard FBA constrained by biomass production was unable to recapitulate measured *in vivo* fluxes through the TCA cycle (Figure S3B). To obtain a more accurate objective function, we integrated information from the metabolomics dataset. We reasoned that a statistically significant change in the concentration of a metabolite between two consecutive time points could only happen if the metabolite was either net-produced or net-consumed between those time points. Therefore, we added a system boundary for the production or consumption of the relevantly changed metabolites as an additional objective function at each time point and simultaneously maximized biomass as well as production or consumption of the relevant metabolites (Figure 5). We called this method Metab_FBA (Supplementary Table 7) using the combined objective function, and referred to the method where only the conventional objective function was used as Standard FBA (Supplementary Table 1). The new metabolomics-integrated objective function provides the model with a guideline for production and consumption of metabolites, without supervising the possible solutions reached by the model, therefore the predictive value of the re-optimized objective function is 2-fold. Firstly, it provides quantitative information about the most affected fluxes when producing or consuming a measured metabolite, and secondly, it provides predicted fluxes for all reactions that produce or consume unmeasured metabolites.

The addition of metabolomics data to the objective function does not dramatically change the enrichment of metabolic pathways represented, although there are differences in rank (Figure S3D and Supplementary Table 2). Previous approaches to incorporate -omics data with FBA have been validated by confirming that the overall variability of flux predictions - the sum of the range of flux variabilities for each reaction–is reduced (e.g., Kleessen et al., 2015). A large variability usually represents a large solution space that is reduced by applied constraints. We have similarly ascertained the variability range for our flux predictions (Figure S9A) and confirm that the inclusion of the transcriptomics data as constraints does reduce the flux variability ranges, in line with what has been previously reported, but inclusion of metabolomics data alongside biomass as the objective function on top of the transcriptomics-derived constraints does not appreciably reduce the flux variability further. This is to be expected, since the method of integration -as an additional component of the objective function- does not aim to constrain the solution space, but rather helps to guide the selection of an optimal solution within the solution space to be more physiologically accurate. We furthermore compared our approach to an approach which incorporated the metabolomics data as constraints on the production or consumption of metabolites rather than as additional information in the objective function. We observed that incorporation as constraints, in some cases, narrows the solution space too much, due to incompatible metabolomics and transcriptomics constraints, leading to the inability of the model to generate biomass under that setup (Figure S9B). This highlights that incorporation of the metabolomics information as objective function gives the maximal flexibility to the system to find the best possible solution maximizing congruence between transcriptomics and metabolomics datasets.

### Case Study: Using Two Objective Functions to Predict Metabolic Fluxes Through the TCA Cycle in Aging Animals

**Figure 6:**
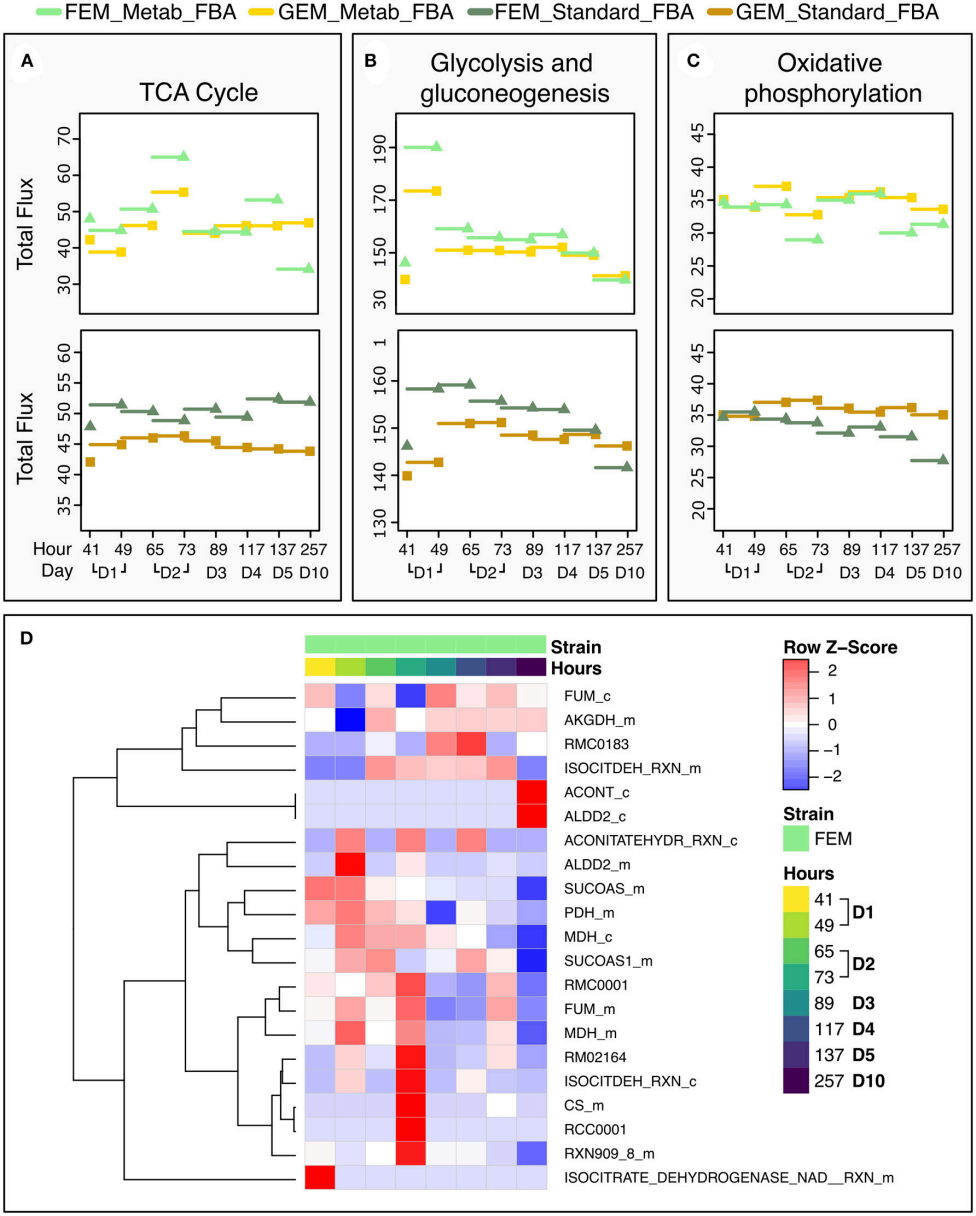
Dynamic changes in pathway fluxes over time. **(A–C)** Dynamic Changes in Fluxes as a function of age, grouped by pathways. These plots show the magnitude of summed flux values at different timeframes per strain and pathway for the TCA cycle **(A)**, glycolysis **(B)**, and oxidative phosphorylation **(C)**. As before, the fluxes are plotted against the timepoint that provided the transcriptomics data. Example the flux at 137 h is the flux computed for the timeframe between 117 and 137 h. **(D)** Heat Map of the Fluxes catalyzed by reactions related to the TCA cycle in FEM animals at day 1 (41 and 49 h), day 2 (65 and 72 h), day 3 (89 h), day 4 (117 h), day 5 (137 h), and day 10 (257 h). Note that time point 97 is not shown as there were no significant metabolic differences at that time point. Greater flux values are indicated in red, lower in blue. As the underlying model represents bidirectional reactions, in some cases, fluxes in the reverse direction are encoded by negative flux values. The heat map here shows the absolute magnitude of the fluxes; the direction of the fluxes and the reactions are given in Supplementary Tables 1, 7. All reactions and metabolites used by the WormJam model are provided in Supplementary Tables 12, 13.

To test the performance of the two FBA variants, we focused on the TCA cycle because it is conserved between *C. elegans* and mammals, and well-annotated in *C. elegans* metabolic reconstruction models. Metab_FBA provides different fluxes than standard FBA in several aspects of central metabolism (Figures 6A–C). First it predicts a peak in glycolysis during day 1 and in TCA cycle during day 2, which is also evident in the heatmap of TCA cycle fluxes (Figure 6D). Within the same time frame, oxidative phosphorylation shows a compensatory decrease, which is not evident using standard FBA (Figure 6C). Interestingly, in FEM animals but not GEM, Metab_FBA predicts a sharp decline in TCA cycle total flux between days 4 and 5 (subsequently referred to as time frame “days 4-5”) and between days 5 and 10 (subsequently referred to as time frame “days 5-10”), and this sharp decrease in metabolic function is not predicted by standard FBA.

**Figure 7:**
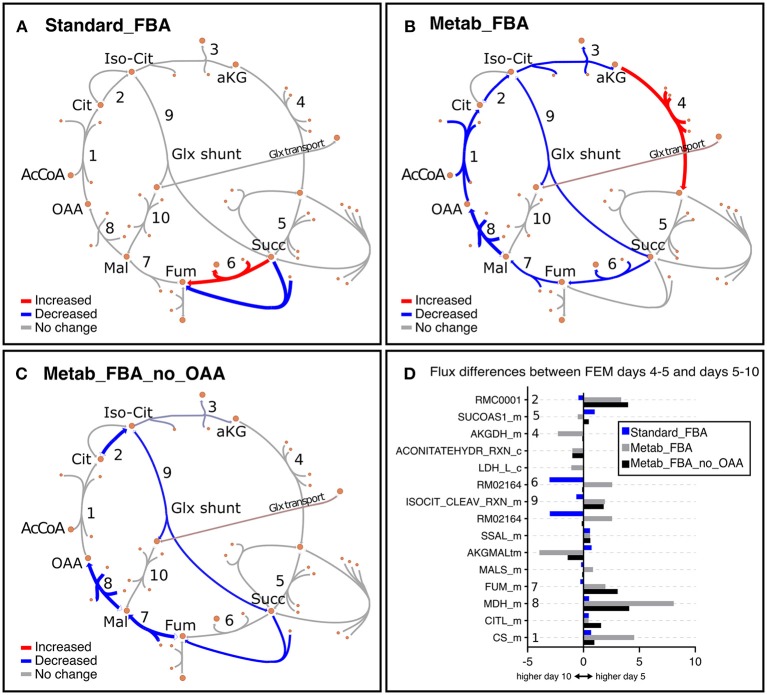
Representation of metabolic fluxes through the TCA cycle in FEM animals using three different objective functions. **(A–C)** TCA cycle reactions represented with the *Escher* tool (King et al., 2015) as a comparison between FEM data for the timeframes at days 4–5 (117–137 h) and days 5–10 (137–257 h) for FBA objective function variants Standard_FBA **(A)**, Metab_FBA **(B)**, and Metab_FBA_no_OAA **(C)**, which does not include **(A)** or includes information regarding all quantified metabolites **(B)** or all except oxaloacetate **(C)** in their respective objective functions. The maps have been colored in Inkscape and colors represent the difference in the fluxes between the two time points, where red is increased and blue is decreased. The weight of the arrows has been adjusted to indicate the magnitude of the change. **(D)** The differences between the two time frames are illustrated per reaction for each of the three FBA variants. The reaction identifiers from the WormJam model shown in **(D)** are illustrated in Figure 2A as follows: R_RMC0001: isocitrate hydro-lyase (reaction 1); R_ACONITATEHYDR_RXN_m: aconitase - isocitrate hydrolase (reaction 1a); RMC0001: Cytosolic isocitrate hydrolase (reaction 2) ; AKGDH_m: Mitochondrial 2-oxoglutarate dehydrogenase (reaction 3); RXN909_8_m: Mitochondrial succinate dehydrogenase (reaction 4); RM02164: Succinate:ubiqui oxidoreductase (reaction 5), FUM_m: Mitochondrial fumarate hydratase (reaction 6), MDH_m: Mitochondrial malate dehydrogenase (reaction 7), CITL_m: Citrate lyase (reaction 8), ISOCITDEH_RXN_m: Mitochondrial isocitrate dehydrogenase (reaction 9). The reactions represented in TCA diagrams have been labeled with appropriate numbers in the Y axis. All other reactions and metabolite identifiers are listed in Supplementary Table 10.

As noted earlier, we noticed that GEM mutants were incompletely penetrant, thus some animals developed a rudimentary gonad and were able to produce progeny. To eliminate potential confounding effects of the small number of progenies, we focused our analysis primarily on FEM animals, which are 100% sterile. In line with our previous observation that Standard FBA does not accurately recapitulate *in vivo* measured fluxes (Figure S3B), we observed that Standard FBA predicted that most fluxes across the TCA cycle remained unchanged between days 5 and 10 (Figure 7A). However, we know this cannot be correct as we had demonstrated that many TCA-related metabolites change with age (Figure 4 and Figure S8). When we included our metabolomics data as part of the objective function, the model predicted a general decrease in fluxes through the TCA cycle in agreement with the measured changes in metabolites (Supplementary Table 10 and Figure S8). These improved predictions should thus provide more confident grounds for hypotheses surrounding metabolites that were not measured.

We looked into oxaloacetate in more detail because fluxes through this metabolite were predicted to be the most depressed with age. Metab_FBA predicted that fluxes through the reaction MDH_m, which represents the conversion of malate to oxaloacetate by malate dehydrogenase in the mitochondria, was dramatically reduced from days 4–5 and days 5–10 in FEM (reaction 8 in Figures 7B,D). At the same time, CS_m, which represents the conversion of oxaloacetate to citrate in the mitochondria, was also substantially reduced (reaction 1 in Figures 7B,D). To get a measure for the net production of oxaloacetate in the mitochondria, we calculated the difference between the sums of all mitochondrial oxaloacetate catabolic and anabolic reactions for each timeframe. This returned values of 7 on days 4–5 and 4 on days 5–10 (a 43% reduction) (Supplementary Table 10). Thus, the Metab_FBA model predicts that net production of oxaloacetate in the mitochondria drops after day 4. Although we did not provide specific instructions as to what solution to find, in this model the objective function contained the information that oxaloacetate levels dropped between these two timeframes. One possibility is that this predicted flux is based solely on the direct measurements of oxaloacetate, and if so, this may indicate that the inclusion of this information was disproportionately biasing the data. To rule this possibility out, we re-executed Metab_FBA without measurements for oxaloacetate, and termed this variant, Metab_FBA_no_OAA (Supplementary Table 8). In this model (Figures 7C,D), we found that fluxes through MHD_m (reaction 1 in Figure 7C) remained the most depressed from days 4–5 to days 5–10 and net production of oxaloacetate dropped by 25% (from an 8 on days 4–5 to 6 on days 5–10). This indicated that even without additional information for this specific metabolite, the model still correctly predicted a substantial drop in oxaloacetate production.

The striking difference in the fluxes within the TCA cycle affecting oxaloacetate between days 4–5 and 5–10 suggested that oxaloacetate may be a limiting metabolite when animals reach middle age. The accuracy of a model is generally tested by its ability to predict gene essentiality or metabolic functionalities (Opdam et al., 2017). In the case of aging animals, survival is a more appropriate phenotype to use for benchmarking purposes. We reasoned that if a metabolite becomes limiting with age, then supplementation of the metabolite in the diet should increase survival, or in other words, extend lifespan. Consistent with this prediction, while supplementation of most TCA metabolites either does not affect lifespan, or has only a small effect (Edwards et al., 2013, 2015), oxaloacetate extends lifespan by 25% and it depends on both AMP-activated protein kinase (AMPK) and insulin signaling (Williams et al., 2009). This illustrates how the new objective function in Metab_FBA outperforms a standard objective function based on growth by providing more accurate predictions about a key metabolite. The fact that oxaloacetate supplementation has been shown to have such an important impact on lifespan also indicates that dysfunctional mitochondria is not just a co-morbidity related to age deterioration, but rather that it is one of the drivers of the aging process.

**Figure 8:**
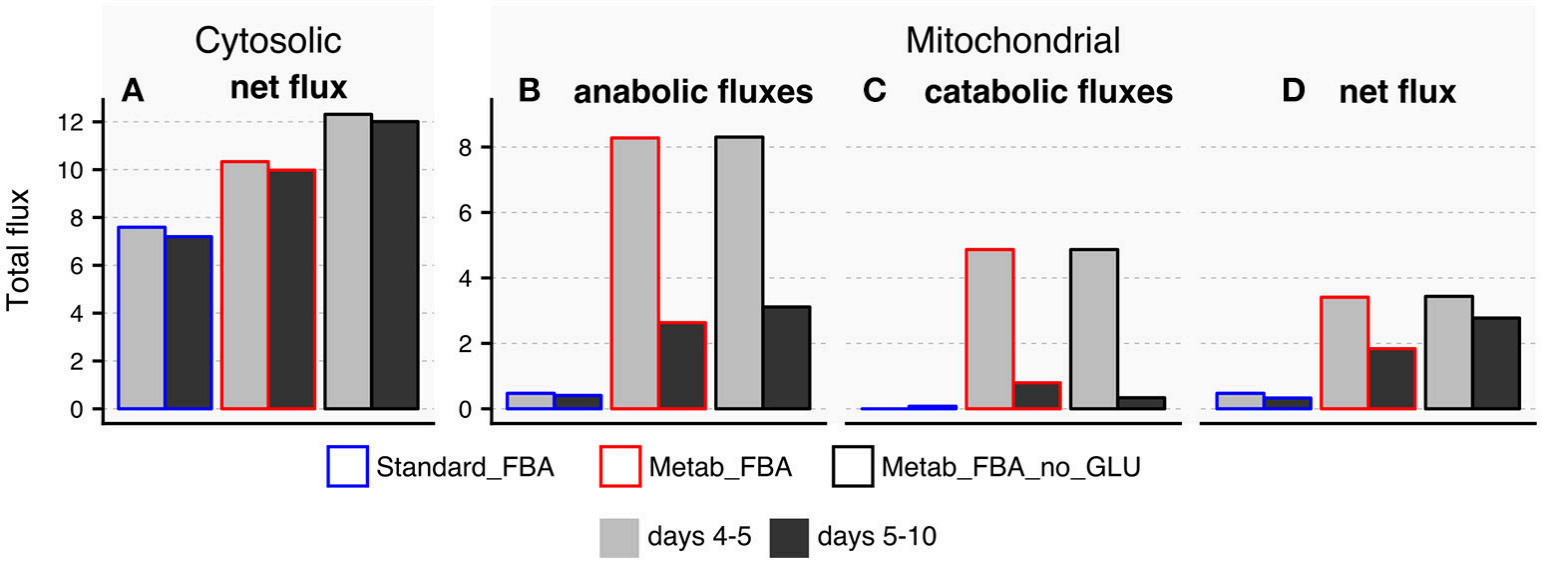
Predicted exchange fluxes for glutamate inside the mitochondria and cytosol by three different FBA models. All comparisons refer to reactions that connect glutamate to the TCA cycle in days 4–5 or days 5–10 FEM samples using three different FBA models: Metab_FBA, which includes information for all measured metabolites; Metab_FBA_no_GLU, which includes information for all measured metabolites except for glutamate; and Standard_FBA, which does not include metabolite measurement information. **(A)** Difference between cytosolic consumption and production. **(B)** Fluxes that produce glutamate within the mitochondria **(C)** Fluxes that consume glutamate inside the mitochondria **(D)** Difference between mitochondrial production and consumption per day per model. All data is available in Supplementary Table 11.

The TCA cycle is a source of energy but it can also produce and consume amino acids such as glutamate and aspartate (reactions 10 and 11 in Figure S3A). We therefore hypothesized that the age-related disruption of the TCA cycle could account for alterations in these amino acids with age. Because we did not directly measure aspartate, we focused our analysis on glutamate, which significantly decreased with age (Figure 4). The fluxes through reactions that connect glutamate to the TCA cycle are listed in Supplementary Table 11. Fluxes predicted by standard FBA are either inactive or unchanged from days 5 and 10 in FEM animals (Figure 8), and therefore conventional FBA modeling does not account for our *in vivo* measurements of glutamate. When using the modified objective function, Metab_FBA predicts that several fluxes that connect TCA metabolites with glutamate are substantially altered (Figure 8). First, although fluxes that lead to glutamate production in the cytosol remain unchanged (Figure 8A), all fluxes inside the mitochondria that contribute to production or consumption of glutamate were decreased (Figures 8B,C). To illustrate, the sum of total fluxes inside the mitochondria dropped from 3.4 to 1.8 according to Metab_FBA, whereas Standard FBA predicted a reduction from 0.47 to 0.33 from days 5 to 10 (Supplementary Table 11 and Figure 8D). To specifically rule out that the optimized objective function is not biasing the results by forcing fluxes through glutamate, we asked if the model arrives at a similar solution in the absence of direct prior information about glutamate. In this *in silico* experiment, fluxes were re-calculated using a modified objective function that included all measured metabolites except for glutamate (Metab_FBA_no_GLU, Supplementary Table 9). Eliminating this information, however, did not significantly change the overall results and predicted that the net fluxes through the mitochondria decrease from 3.4 to 2.7, which is a smaller reduction than Metab_FBA, but still much higher than standard FBA (Supplementary Table 11 and Figure 8), indicating that the model's predictive capacity is robust. Given that fluxes leading to catabolism of glutamate are predicted to sharply decrease with age, a plausible hypothesis is that depletion of glutamate in old animals is caused by a failure of the TCA cycle to replenish glutamate. This model is likely to be correct for two reasons. First, as pointed out earlier, the TCA cycle appears to be dysfunctional in old animals. Secondly, this prediction is consistent with fluxes measured *in vivo* using labeled carbon sources. Schrier Vergano et al. (2014) found that upon knock-down of components of ETC complexes I to IV as well as in TCA-cycle defective *idh-1* mutants, glutamate become depleted in worms (Figure S8), connecting a malfunctioning TCA cycle with cellular AAC content.

## Discussion

### Potential Drivers of the Differences in Sample Variability Between Metabolomics and Transcriptomics Data

One possible reason for the observed discrepancy in sample variability between transcriptomics and metabolomics (Figure 2) is that the targeted metabolomics assay we used covers a small subset of metabolites of central importance, which are therefore likely to be highly conserved across strains under similar environmental conditions. Conversely, transcriptomics samples thousands of transcripts and is therefore likely to be much more sensitive to strain-to-strain differences. We might thus hypothesize that the metabolomics data should show more between-strain differences if we had used an untargeted metabolomics assay in which the dataset comprised all metabolites. An alternative explanation is that the most important source of metabolites in worms is the intestine, whereas the germline may be depleted of metabolites but enriched in transcripts. Thus, the different germline composition of GEMs and FEMs may explain the divergent trajectories. However, we obtained similar results when we excluded genes shown to be enriched in the germline (data not shown), making this less likely. Finally, there are several layers of regulation upstream of metabolites and the misalignment between metabolomics, and transcriptomics may indicate an important layer of post-transcriptional control of metabolism.

### The Middle Age Switch to Produce Polyamines and Ketone Bodies May Be the Consequence of Reduced Food Intake With Age

We report for the first time a large increase in polyamines which happens after day 5 of adulthood. The levels of the metabolite spermidine were highly variable in our dataset and thus we could not detect a significant change in levels across time. However, as shown in Figure S7, spermidine synthase, which catalyzes the biosynthesis of spermidine, is upregulated. Supplementation of putrescine and agmatine increases lifespan in worms by 10–20% (Edwards et al., 2015) whilst the role of cadaverine remains unexplored. Spermidine has been associated with lifespan extension by activating autophagy (Eisenberg et al., 2009; Minois, 2014). Autophagy is a well-described fasting response that recycles cellular components to restore the energy balance. It is interesting to note that the levels of 3-hydroxybutyric acid (3HBA) behave similarly to the polyamines. 3HBA is metabolized from ketogenic amino acids, and/or lipids and it is a component of ketone bodies, which supply energy during periods of fasting (Veech et al., 2017). This suggests that 10 day old animals have reduced food intake, perhaps as a consequence of well-described loss of pharyngeal pumping activity with age (Russell et al., 2017). At the same time, we observe significantly increased levels of spermidine precursors, thus, polyamine synthesis may be enhanced in day 10 animals so as to stimulate autophagy when nutrient availability from food intake becomes limiting. Autophagy can also mediate the conversion of intestinal biomass into yolk, causing documented early aging pathologies (Ezcurra et al., 2018). Therefore, autophagy can have positive and negative consequences for longevity. The seemingly contradictory nature of these two observations might be further clarified by gaining a deeper understanding of the underlying molecular mechanisms that trigger autophagy and the physiological contexts where it is used. A plausible scenario is that the increase in polyamines that may result from reduced food intake at day 10, may promote a switch in autophagy away from lipoprotein pool production into a survival strategy aimed at coping with malnutrition, which may be beneficial for survival.

### Limitations of Metab_FBA and Outlook

We have presented the first study of aging in normal-lived *C. elegans* to use FBA together with a time-resolved transcriptomics and metabolomics dataset. While we used existing strategies for the transcriptomics integration, the strategy we used to integrate the time-resolved metabolomics data with the objective function of the FBA is novel, and based on our case study of the TCA metabolism we have found that it yields results that are closer to the physiology than what is predicted with transcriptomics data alone.

An important limitation of our method is that incorporation of the metabolomics differences in the objective function without quantitative constraints may result in the model having too much freedom to optimize production or consumption of those specific metabolites and as a result may generate excessive fluxes, although in the right direction. For example, in Figures 6A,B, the model predicts peaks in fluxes through carbohydrate metabolism at 49 h and through the TCA cycle at 73 h. The use of neighboring time points rather than overall trends in metabolomics measurements to calculate differences for incorporation into the model may also lead to an increase in noise (Type 2 errors). To mitigate these problems, we might calculate a linear rate of change for metabolite level changes across a number of time points (as is done, for example, in Bordbar et al., 2017) and use those per-metabolite linear rates of change as constraints on the relevant reactions that are added to the objective function. We plan to test this combined approach in future work. Moreover, the set of metabolites available for the study was limited and may have introduced bias to the results. In future a more comprehensive, untargeted metabolomics assay might be used to obtain a whole-metabolome view on systemic changes due to aging.

Another limitation of our study is the incomplete state of annotation of the model of *C. elegans* metabolism. While the WormJam model represents the consensus of the knowledge represented across all the available published models, it is a work in progress and there are known problems which require further manual curation to resolve (Witting et al., 2018). For example, the annotation of worm-specific metabolites is poor, as is the annotation of pathways involving fatty acids and lipids.

We anticipate that other approaches to changing the objective function will also enhance the use of FBA for the study of aging in *C. elegans*, for example, the addition of *in vivo* measured oxygen consumption and total ATP production data at matching timepoints. We demonstrate that our method is able to accurately predict changes in metabolite levels for which no metabolite information is provided, thus with the addition of relatively easily obtainable data (e.g., metabolites for which good standards exist), our model may be used to predict system level metabolic shifts or changes in metabolites that are more difficult to quantify (i.e., those without appropriate standards).

### Our Data Reveals a Dramatic Drop in Mitochondrial Function With Age and our FBA Analysis Reveals Interconnected Consequences With Other Metabolic Functions

Transcriptomics data indicates that the function of the TCA cycle is gradually reduced and there is a corresponding gradual decrease of mitochondrial oxygen consumption with age (Brys et al., 2010). The production of ATP in older animals has been determined to be 20% that of young adults (Braeckman et al., 2002). However, our metabolomics data shows that TCA intermediates are changed dramatically sometime after day 5 of adulthood (and after day 7, as observed in Gao et al., 2017a). It is not known, however, what triggers the loss of functionality of the mitochondria in normal lived animals with age, but it can be reversed by mutations in AMPK homolog *aak-2*, a master regulator of energy homeostasis (Weir et al., 2017). In *C. elegans*, mitochondrial fusion is necessary but not sufficient for longevity assurance, suggesting that energy must become limiting with age and that stable energy levels must be required to maintain energy consuming processes such as protein folding homeostasis (Chaudhari and Kipreos, 2018).

The mitochondrial free-radical theory of aging–which proposed that free radicals released by mitochondria are the drivers of aging–cannot be correct because removing radicals does not extend lifespan (Honda et al., 2008). Recently, an alternative idea has been proposed where free radicals cause local damage to the mitochondria, causing a drop in energy production (Chaudhari and Kipreos, 2018). Free reactive oxygen species (ROS) can also have a healthy hormetic effect on mitochondria (Ristow and Zarse, 2010) so for this theory to be correct, ROS action has to be threshold dependent. One potential explanation for our observations of the loss of TCA cycle function is that such a threshold has been crossed after day 7, precipitating the loss of mitochondrial function. In support of this hypothesis, we also observed a drop in aconitate levels, which is synthesized by aconitase. The activity of this enzyme is sensitive to ROS because it undergoes oxidative modification and inactivation during aging and in certain oxidative stress related disorders (Lushchak et al., 2014). Interestingly, we observed that aconitate levels drop earlier than oxaloacetate levels, suggesting that ROS may precipitate TCA cycle dysfunction. Another possible explanation for our observations is that altered mitochondrial dynamics cause local imbalances between the levels of substrates and products, which could initiate aberrant feedback loops or reduce enzyme efficiency. A completely different explanation is that by day 10 many of the described comorbid pathologies have already reached their maximum levels (Ezcurra et al., 2018) pushing the limits of the system into collapse. Because the collapse of mitochondrial function is evident in the metabolomics data and not in transcriptomics, a post-transcriptional switch may be required. The addition of proteomics data would help to address this question, and is indicated for future work.

Overall, we observe that the use of FBA with metabolomics as part of the objective function provided accurate predictions with regards to the most affected reactions of the TCA cycle with age. Specifically, using the combined objective function accurately predicted that oxaloacetate is the metabolite that becomes most limiting with age, a prediction that matches the observation that it is the single most effective TCA cycle intermediate in extending lifespan upon supplementation (Williams et al., 2009). Oxaloacetate supplementation elevates the levels of NAD+ and restores redox balance, acting through sirtuins and AMPK (Roth and Ingram, 2016). In mouse models of stroke, oxaloacetate administration has been reported to reduce neural damage and traumatic brain injury (Roth and Ingram, 2016). Although the role of oxaloacetate as a healthspan modulator requires more scrutiny, a product containing oxaloacetate is already marketed for human consumption (www.benegene.com). There are also NAD+ synthesis stimulation therapies being marketed for human consumption (e.g., Basis by Elysium). Our model also predicts that the TCA cycle can have an impact on the fluxes of amino acids such as glutamate. Other more extensive predictions that relate the TCA cycle to lipid metabolism will be possible when the metabolic reconstruction model becomes extensively annotated for these pathways.

## Author Contributions

OC and JH designed the study. JH, JP, BV, and OC performed the analyses. AM, BV, and SM performed experiments. BV, SM, and AM arranged the Figures. JH and OC wrote the manuscript and all authors contributed to editing.

### Conflict of Interest Statement

The authors declare that the research was conducted in the absence of any commercial or financial relationships that could be construed as a potential conflict of interest.
