# Supplementary material for: Multi-Omics and Genome-Scale Modeling Reveal a Metabolic Shift During C. elegans Aging
Source: Front Mol Biosci. 2019 Feb 6;6:2. doi: 10.3389/fmolb.2019.00002 (PMC6372924; doi:10.3389/fmolb.2019.00002)
Supplement: Supplementary file 1 [file Data_Sheet_1.pdf]

Supplementary Material

Multi-omics and genome-scale modeling reveal a metabolic shift during *C. elegans* aging.

Janna Hastings[1]; Abraham Mains[1]; Bhupinder Virk[1]; Nicolas Rodriguez[1]; Sharlene Murdoch[1]; Juliette Pearce[1]; Sven Bergmann[2]; Nicolas Le Novère[1]; Olivia Casanueva[1*].

[1.] Babraham Institute. Babraham Research Campus CB22 3AT, Cambridgeshire, UK

[2.] Department of Computational Biology, University of Lausanne, Switzerland

[*] correspondence: olivia.casanueva@babraham.ac.uk

SUPPLEMENTARY METHODS

1. Determination of age-associated metabolites (Additional information)

As described in Thevenot et al., the ropls R package implements the PLS(-DA) approaches with the original version of the algorithm (Wold et al., 2001) and includes the R2 and Q2 quality metrics which estimate the significance of the model by permutation testing, the permutation diagnostics, the computation of the VIP values, the score distances to detect outliers, as well as many graphics (scores, loadings, predictions, diagnostics, outliers, etc.).

The ropls.opls function was executed with the normalized metabolomics dataset and sample age in hours as the response to be modeled. Model output was as follows:

```
47 samples x 105 variables and 1 response

standard scaling of predictors and response(s)

      R2X(cum)    R2Y(cum)    Q2(cum)     RMSEE       pre ort     pR2Y  pQ2

Total 0.49        0.978       0.921       8.8         3    0      0.05  0.05
```

RMSEE is the square root of the mean error between the actual and the predicted responses. Basically, the summary statistics show that the model provides good predictions (Q2 is close to 1) but

with the proviso that the underlying data are noisy (RMSEE is large). R2X (respectively R2Y): is the cumulative percentage of predictor (respectively response) variance explained by the full model. Q2 is the cumulative predictive performance of the model estimated by cross- validation. Pre is the number of components required to achieve predictive performance, ort is the number of orthogonal components (not relevant for this scenario).

Metabolites were considered to be age-associated if their Variable Influence on Projection (VIP) given by the model was greater than 1.

2. **Determination of longevity modulating metabolites**

To determine which metabolites were known to be longevity modulators in *C. elegans*, i.e. those that extended lifespan when supplemented to *C. elegans*, we consulted the DrugAge database of known longevity modulators (Barardo et al., 2017) and our own examination of the published literature. Note the DrugAge database also includes metabolites that are detrimental to longevity, and in some cases conflicting results were found in DrugAge (e.g. glucose, for which DrugAge contained a record of an assay in which glucose supplementation had extended longevity, and several records of assays in which glucose supplementation had reduced longevity). In general, we went with the majority of reports in DrugAge - for example, in the case of glucose, we went with the consensus of no positive effect.

3. **Flux variability analysis**

Flux variability analysis (FVA) evaluates, for each reaction, the maximum and minimum fluxes that would be possible with the same objective value. We evaluated the flux variabilities consistent with the objective by using the COBRAPy "cobra.flux_analysis.flux _variability_analysis" implementation as a second step after obtaining the optimal value for the objective function. Flux variability gives a maximum and a minimum flux for each reaction; to obtain the flux variability range we calculated (maximum - minimum).

4. **Pathway analysis**

Pathway enrichment was performed using R package Gage (Luo et al., 2009) on the transcriptomics and metabolomics data for FEMs between day 5 and day 10, then visualized using the R Package Pathview (Luo and Brouwer, 2013). This allowed us to extract enriched pathways, and visualize directional changes in the same schematic of both transcript and metabolites.

5. **Flux-based pathway enrichment analysis**

To investigate which pathways are most responsible for the changes observed between times, we used a binomial test to identify overrepresentation of pathways in the 10% most different reactions in FEMS between days 5 and 10 for Standard FBA and days 4-5 and 5-10 for Metab_FBA, tested against a background list of all reactions. We also identified fold enrichment for each pathway as described in (Zhang et al., 2005).

6. RNA-sequencing library preparation.

Samples of at least 1000 worms were washed 3X in M9 by settling then snap-frozen in Trizol and kept at -80C until library-prep. Samples were freeze-thawed 10 times to break worm cuticle and RNA was precipitated using chloroform-isopropanol. RNA was treated with 10U DNase I (Roche #04716728001) for 20 minutes at 37°C and purified on Qiagen RNeasy columns (QIAGEN 15596-026). Libraries were prepared using combined protocols from SuperScript II Reverse Transcriptase (Invitrogen 18064-014), NEBNext mRNA Second Strand Synthesis Module (E6111), and NEBNext Ultra II DNA Library Prep Kit for Illumina (NEB-E7645). Half reactions were used for all steps except the final PCR amplification. Briefly, Oligo d(T)25 beads (NEB-S1419S) were washed and resuspended in 2X RNA Binding Buffer (2 M LiCl , 80 mM Tris HCl (pH 7.5), 4 mM EDTA , 0.2% Triton X-100) in a 1:1 ratio and 500ng of total RNA was added. RNA was denatured by incubating at 65C for 5 minutes and mRNA was allowed to bind to beads. Beads were washed twice in Wash Buffer (150 mM LiCl , 20 mM Tris HCl (pH 7.5), 1.0 mM EDTA , 0.01% Triton X-100) and mRNA eluted in 10 mM Tris Buffer by incubating at 80C for 2 minutes. An equal volume of Binding Buffer was added, washed once more, and the mRNA was eluted into a First Strand Synthesis Reaction Buffer containing 4μl 5X First-Strand Buffer (Invitrogen), 1μl 50μM Random hexamers (Invitrogen N8080127), 0.5μl 10mM dNTP mix, and 1.5μl 0.1M DTT. 0.5μl RNaseOUT (Invitrogen 10777-019), 2.5μl 0.1μg/μl Actinomycin D (Sigma A1410), and 0.5μl SuperScript II Reverse Transcriptase were added and samples were incubated in a thermal cycler for 10 minutes at 25°C, 15 minutes at 42°C, and 15 minutes at 70°C. Second strand synthesis was performed immediately using the NEBNext mRNA Second Strand Synthesis Module. Libraries were then generated using the NEBNext Ultra II DNA Library Prep Kit. For PCR library enrichment, 13 cycles of amplification were performed. Library quality was assessed on a Bioanalyzer High Sensitivity DNA Chip (Agilent 5067-4626) and concentration was determined using KAPA Library Quantification Kit (KK4824). Libraries were sequenced on an Illumina HiSeq 2500 system by the Babraham Sequencing Facility.

7. RNA-Seq data preparation and normalization

Libraries were trimmed to 50bp with trim galore v0.4.4 using default parameters. Trimmed data was mapped to the *C. elegans* WBCel235 genome assembly using hisat2 v2.1.0 guided by splice junctions taken from Ensembl v75. Mapped positions with MAPQ < 20 and all non-primary alignments were discarded before the data was analyzed. All libraries were mapped as non-directional single-end. Only exactly overlapping reads were assigned to a gene and transcript-isoforms were merged. Raw counts were generated using Seqmonk and all subsequent analysis was

performed in R. The DESeq2 package was used to generate normalised counts. Library metrics are presented in Supp. Table 14.

During normalization, DEseq2 accounts for differences in sequencing depth by calculating a size factor using the "median ratio method" (Anders and Huber, 2010). This method mitigates the effect of small numbers of highly and differentially expressed genes by using the median ratio of observed counts to calculate a unique size factor that is then used to scale the libraries. When considering strains that possess substantially different tissue composition this may introduce a significant confounding factor. For example, a gene that is enriched in the germline would produce a high ratio in a germline(+) animal, and a low ratio in a germline(-) animal. Multiplied over many thousands of genes, this would have the effect of increasing the median ratio in germline(+) animals and reducing it in germline(-) animals. Consequently, normalised gene counts for non-germline enriched genes would be artificially deflated or inflated in germline(+) and germline(-) animals respectively. This would particularly affect our GEM samples, which displayed delayed expression of germline-enriched genes.

To determine whether the ratio of germline to somatic gene counts may be affecting our data in this way, we calculated size factors with and without genes that were shown to be enriched in the germline (Han et al., 2017) and which displayed clear reproductive expression patterns and gene ontologies in our data, hereafter referred to as “germline-enriched”. When these genes were excluded, size factors for GEM samples increased at early time points and decreased at later time points (Supp. Figure S1A), mirroring the rising expression of germline enriched genes in this strain. These results confirmed that the ratio of germline to somatic transcripts was having a direct effect on the scaling of our libraries.

To test whether exclusion of germline enriched genes during normalization produced a more accurate estimation of somatic gene counts, we compared gene counts between 49hrs (D1) and 137hrs (D5) timepoints in GEM, where the difference in germline:soma ratio would be the greatest. If the ratio was distorting the data in a consistent manner we would expect non-germline enriched gene counts to be deflated in D5 samples and inflated in D1 samples when counts were scaled to the entire genome. We plotted the counts for non-germline-enriched genes and observed a clear upshift in mean expression in D1 samples when counts were normalised to the entire genome (Figure S1C). When we excluded germline-enriched genes during normalization, this bias was mitigated (Figure S1D). As expected, when making comparison between samples with stable expression of germline-enriched genes (FEM 49hrs vs. FEM 137hrs), this correction had no effect (Supp. Figure S1 E and F). In light of this, we excluded germline-enriched genes when calculating size-factors to produce normalised counts.

SUPPLEMENTARY FIGURES

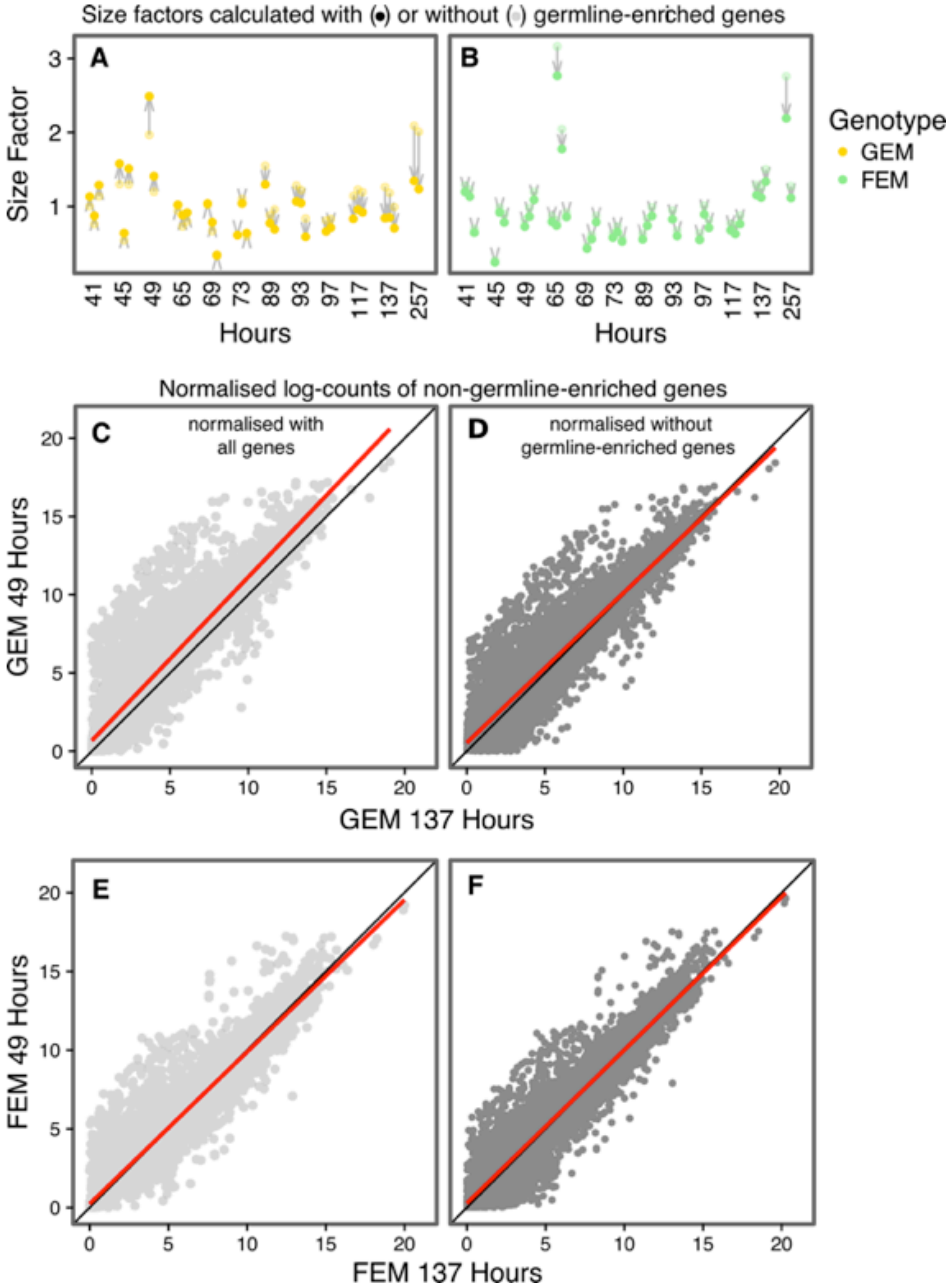

Figure S1. Excluding germline-enriched genes during normalization mitigates bias introduced by different germline:soma ratios.
(A and B) DEseq size factors calculated with (pale dots) or without (dark dots) germline-enriched genes: when germline-enriched genes are excluded size-factors are reduced in germline(+) samples (e.g. GEM 137hrs) and increased in germline(-) samples (e.g. GEM 49hrs). **(C)** When germline-enriched genes are included during normalization, non-germline-enriched gene counts are inflated in germline(-) samples (GEM 49hrs) compared to germline(+) samples (GEM 137hrs). **(D)** Excluding germline-enriched genes during normalization mitigated the over-estimation of gene counts, illustrated by alignment of the fitted linear model (red line) with the expected mean (black line). **(E and F)** Germline-enriched genes were stably expressed across all timepoints in FEM so comparisons between early and late timepoints in FEM were not affected by the germline:soma ratio.

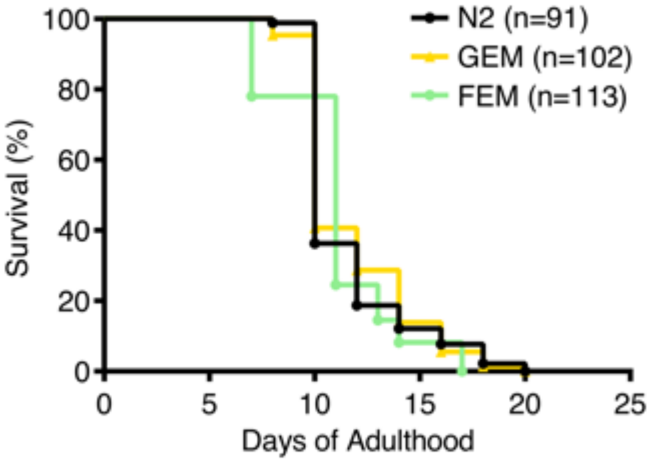

Figure S2. FEM and GEM strains have lifespans comparable to wild type (N2). Lifespan analysis of animals at 25°C. N2, mean lifespan = 11.51 days, GEM mean lifespan = 11.71 days (compared to N2: Log-Rank P = 0.7268 (NS), Wilcoxon P = 0.5979 (NS)), FEM mean lifespan = 11.04 days (compared to N2: Log-Rank P = 0.8502 (NS), Wilcoxon P = 0.2837 (NS)). Data shown are representative of 3 biological replicates.

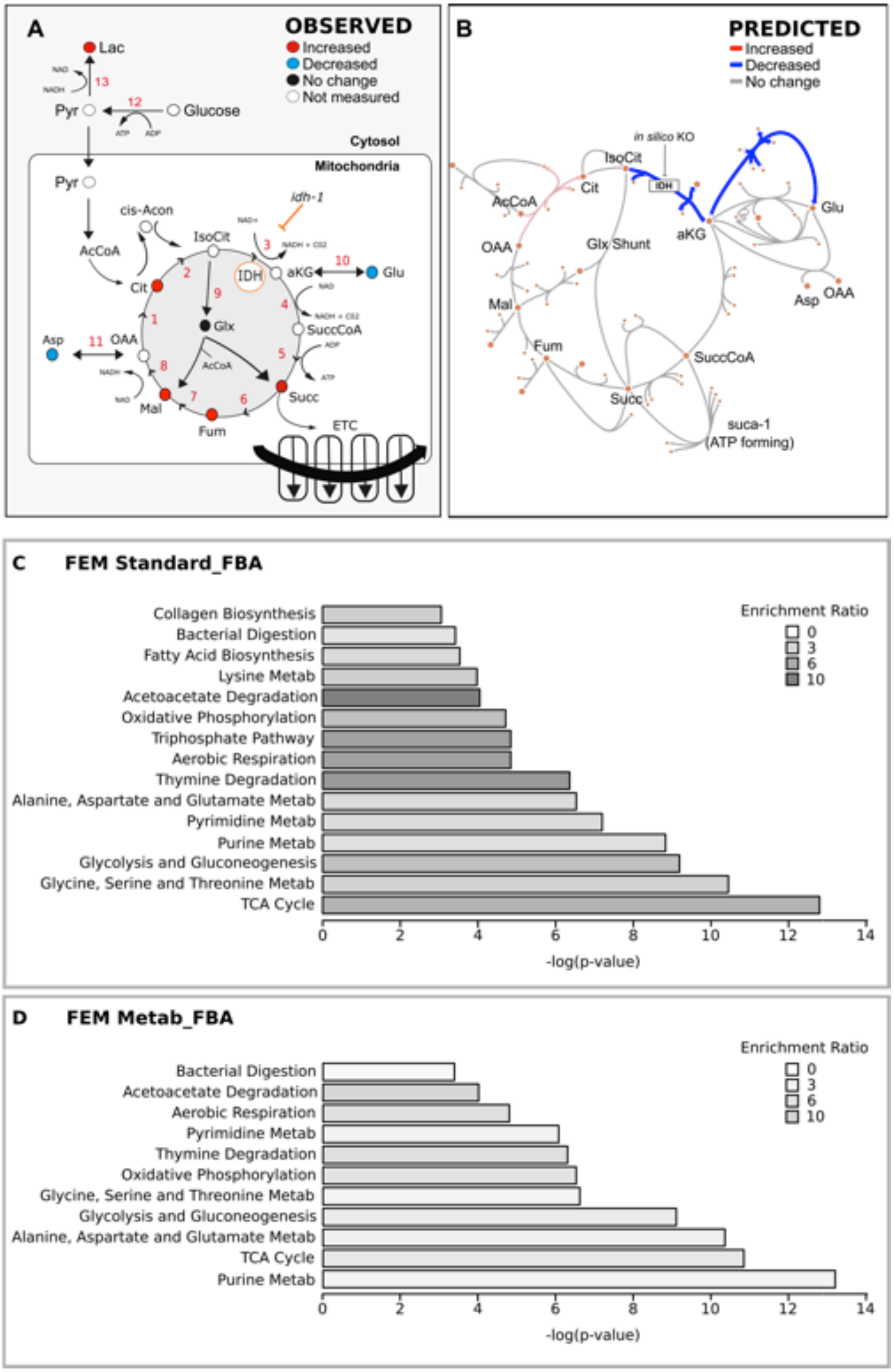

Figure S3. (A-B) ***In vivo*** **measured and FBA predicted fluxes through TCA in mutant strains lacking** ***isocitrate dehydrogenase*** **(C-D) Fold Enrichment for pathways that are differentially represented in flux solutions for FEM.**

(A) Diagram representing the metabolite alterations observed in *idh-1* mutants. Metabolic labeling studied by following the incorporation of 1,6 $^{13}C_2$-glucose ingestion in day 1 animals (Schrier Vergano et al., 2014). Metabolites that are found changed due to altered fluxes in *idh-1* mutants compared to wild type are color coded as follows: blue and red were found to be decreased and increased respectively; black represents metabolites that do not change with age and white represents unmeasured metabolites. Explanation of the TCA cycle: The starting molecule of the TCA cycle is Oxaloacetate (OAA), a 4-carbon molecule which when combined with acetyl-CoA (AcCoA) by citrate synthase forms a 6-carbon molecule, citrate (Cit) (reaction 1) that is further isomerized to Isocitrate (IsoCit) by aconitase (reaction 2). The next two enzymatic reactions result in the loss of 2 carbons in the form of CO2 and in the release of reducing agents NADH and NADPH, which drive ATP synthesis via the electron transport chain (ETC) and fuel lipid synthesis respectively. Isocitrate is the substrate of isocitrate dehydrogenase (IDH) to produce alpha-ketoglutarate (aKG, reaction 3), which is further dehydrogenated into succinyl-CoA (SuccCoA, reaction 4). SuccCoA is metabolized into succinate (Succ), the only ATP releasing step of the TCA cycle (reaction 5). SuccCoA is the substrate of Succinate-coenzyme Q reductase or enzyme complex II, which participates in both TCA and the ETC and generates the reducing agent FADH2 and fumarate (Fum) (reaction 6). Fum is further hydrated into malate (Mal) which undergoes the last removal of protons generating NADH and OAA (reaction 7). Other abbreviations: Cis-aconitate (cis-Acon), Glyoxylate (Glx), Pyruvate (Pyr), Lactate (Lac), Glutamate (Glu), Aspartate (Asp), electron transport chain (ETC). Reactions 1-11 occur inside the mitochondria and 12-13 in the cytosol. **(B)** FBA comparing a wild type young adult with an *idh-1; idh-2 in silico* knock out. Escher was used to build a diagram of the TCA cycle. Blue represents decreased fluxes, gray unchanged, and light pink a small increase in fluxes. **(C-D)** Bar plots showing statistical significance and fold enrichment for overrepresentation of pathways in the top 10% of flux differences in FEM data. (C) without metabolomics data included in the objective of the model for days 5 versus 10, and (D) in days 4-5 time frame versus days 5-10 timeframe when metabolomics data was included in the objective.

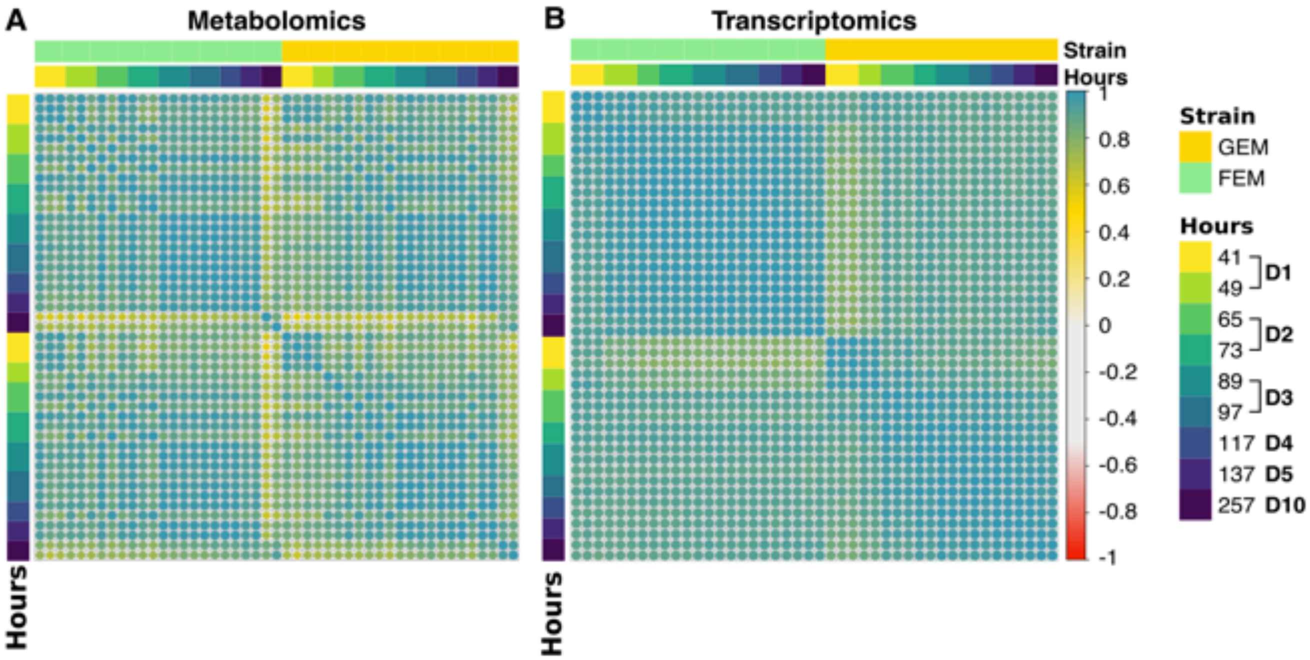

Figure S4. The sample-to-sample correlations are shown for the metabolomics and transcriptomics datasets. (A) shows the metabolomics dataset and **(B)** shows the transcriptomics dataset. Samples are ordered by strain and then by age in hours. These plots illustrate that there is greater variability between samples in the metabolomics dataset, and that the variability is greatest between aged samples in the metabolome but between young samples in the transcriptome. The least variability is within the FEM transcriptome. All samples are positively correlated, as we would expect for a dataset from an isogenic population under controlled conditions. It is clear that the metabolomics dataset has more technical (i.e. between replicate) variability.

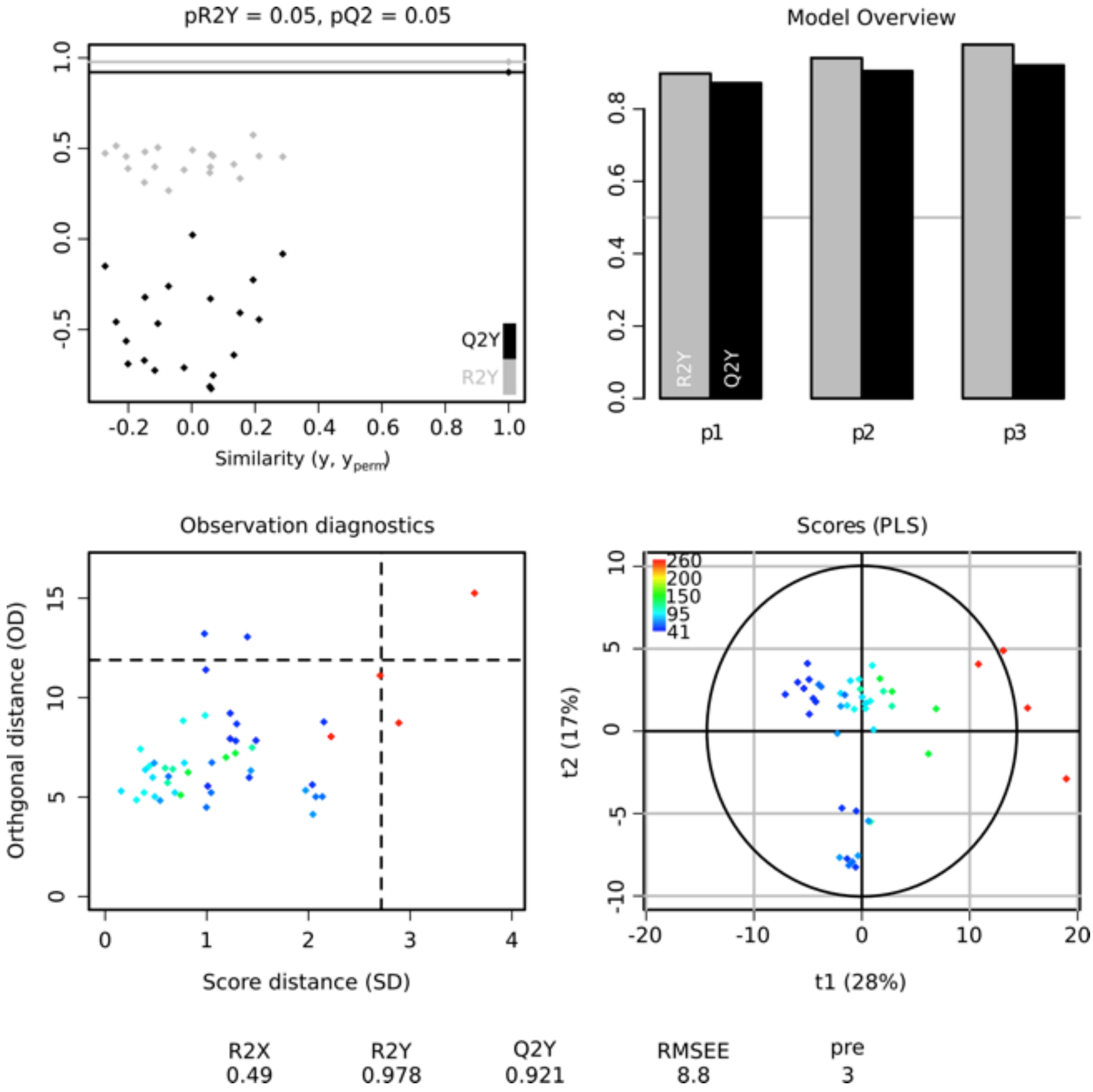

Figure S5. PLS-DA model for ageing metabolome. This Figure is generated by the ropls library when fitting the predictive model to the provided metabolomics dataset. **Top left**: significance diagnostic: the **R2Y** and **Q2Y** of the model are compared with the corresponding values obtained after random permutation of the y response; **Top right**: inertia barplot: the graphic here suggests that 3 components may be sufficient to capture most of the inertia; **Bottom left**: outlier diagnostics; **Bottom right**: x-score plot: the number of components and the cumulative **R2X**, **R2Y** and **Q2Y** are indicated below the plot. In the scores plot, the ellipse corresponds to 95% of the multivariate normal distribution with the samples covariance.

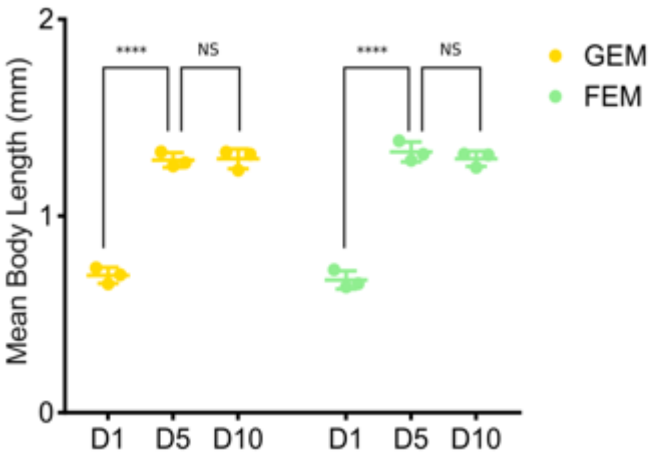

Figure S6. Rate of body size growth with age in wild type *C.elegans*.

Body length significantly increases between Day 1 (41 hours) and Day 5 (137 hours) in both the GEM ($P<0.0001$) and the FEM ($P<0.0001$) strains. Between Day 5 and Day 10 (257 hours) no significant change in body length is observed in either strain. Points plotted represent the mean body length measured from at least 30 animals, biological replicate count $n=3$ for each timepoint, per strain.

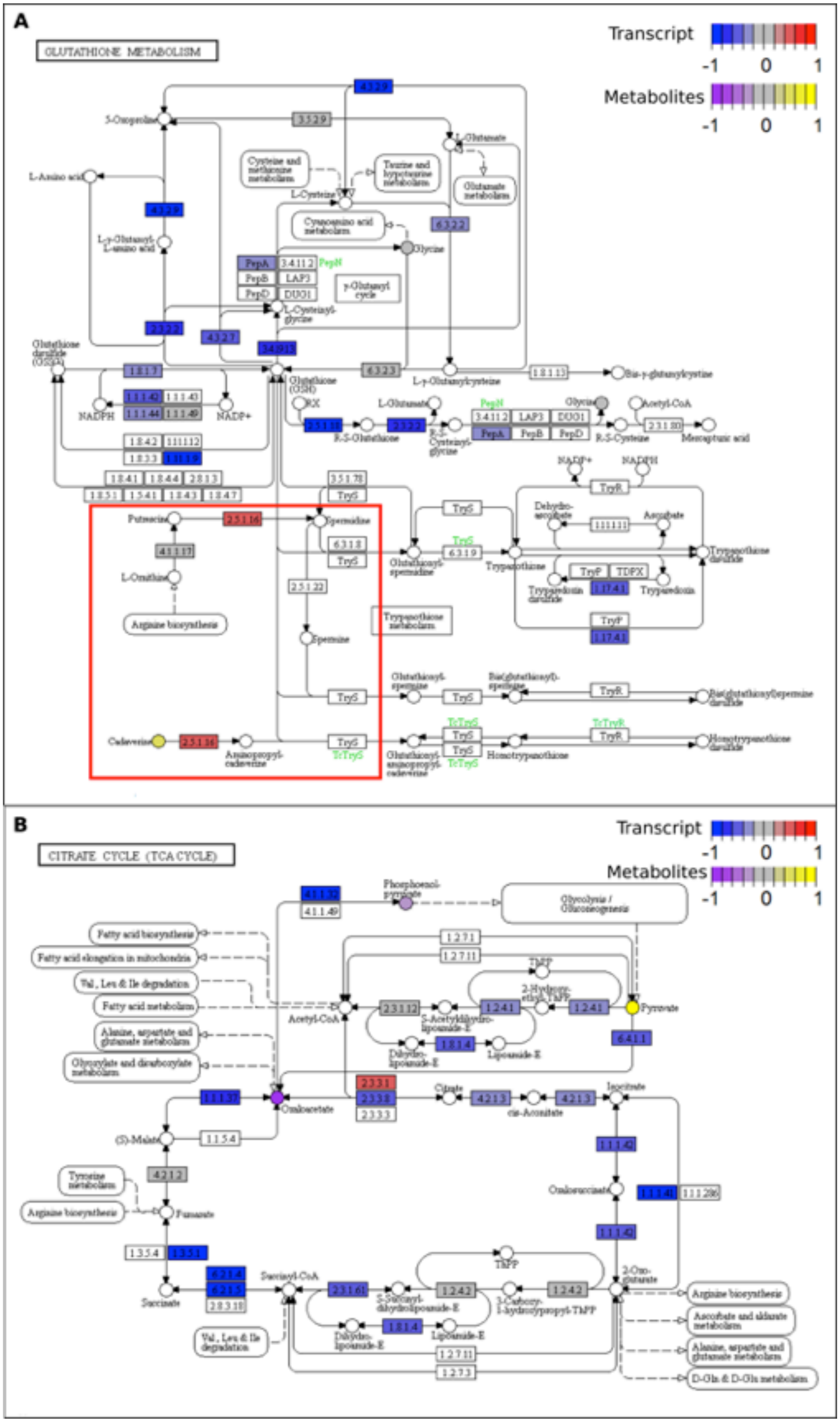
A
GLUTATHIONE METABOLISM
Transcript
-1 0 1
Metabolites
-1 0 1
B
CITRATE CYCLE (TCA CYCLE)
Transcript
-1 0 1
Metabolites
-1 0 1

Figure S7. Pathview comparison of FEM day 5 and 10. (A) The Pathview map of the glutathione metabolism pathway illustrating differences in FEM transcriptomics and metabolomics between days 5 and 10. **(B)** The Pathview map of the TCA cycle illustrating differences in FEM transcriptomics and metabolomics between days 5 and 10.

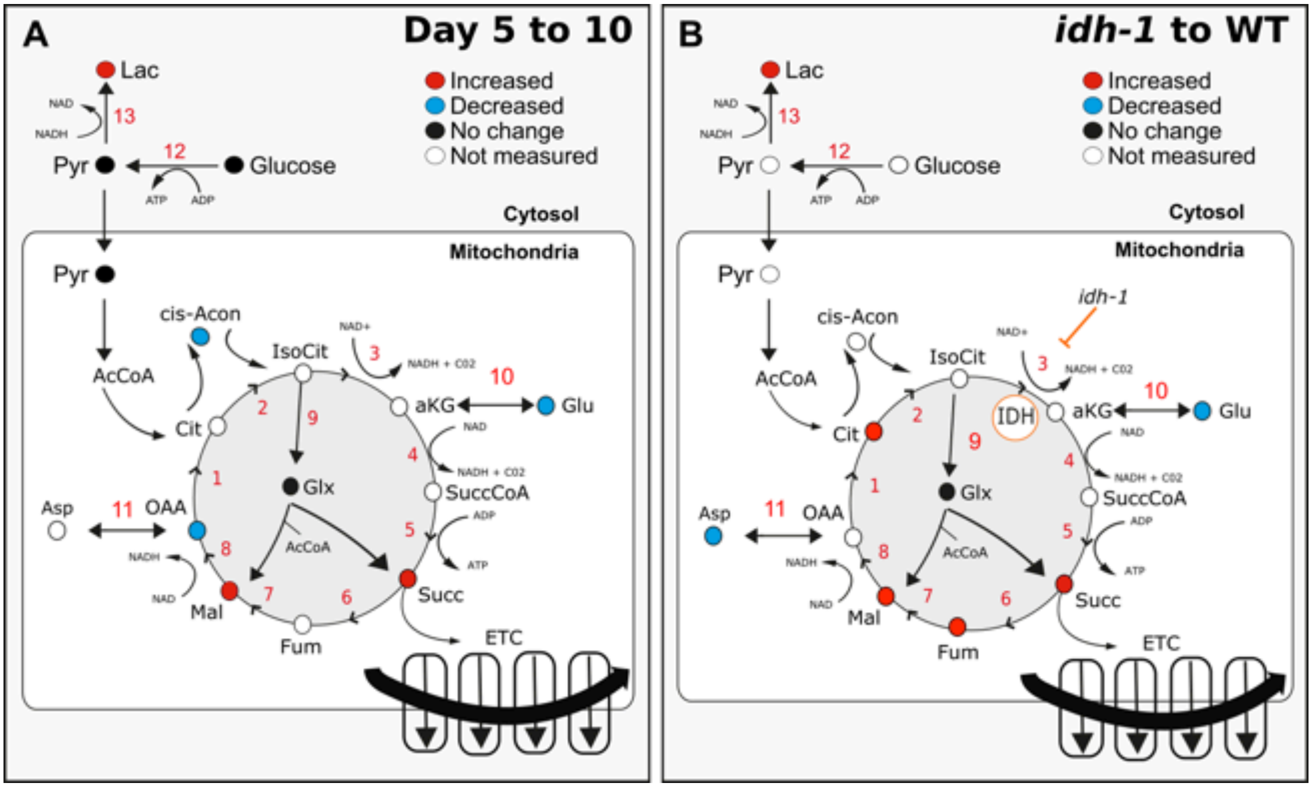

Figure S8. Metabolite measurements show that aged worms have a defective TCA cycle.

Metabolites that are found changed due to altered fluxes in (A) our dataset of old worms, and (B) *idh-1* mutants compared to wild type from (Schrier Vergano et al., 2014). The metabolites are color coded as follows: blue and red were found to be decreased and increased respectively; black represents metabolites that do not change with age and white represents unmeasured metabolites.

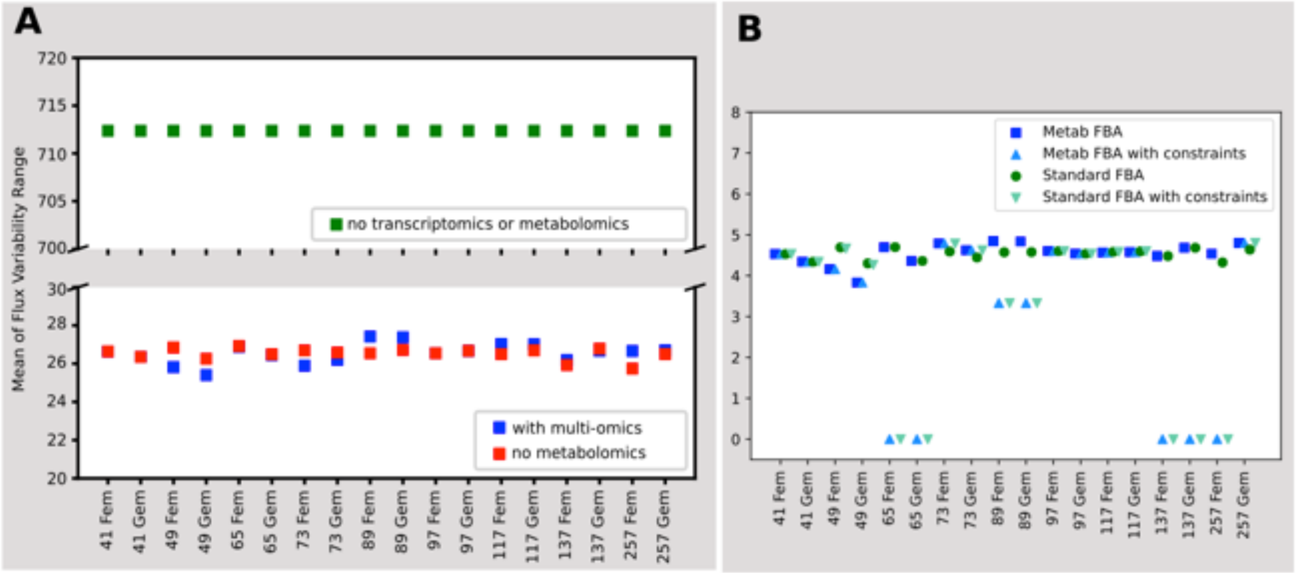

Figure S9. Flux Variability Analysis of the Transcriptomics, Metabolomics + FBA predictions.

(A) We conducted a Flux Variability Range analysis to validate the multi-omics inclusion with FBA, by computing the flux variability for each reaction in the model under three conditions: (1) with no -omics data; (2) with transcriptomics data only (no metabolomics); and (3) with both transcriptomics and metabolomics data (with multi-omics). The mean of the flux variability range (variability.maximum - variability.minimum) is taken across all the reactions in the dataset and plotted per time point and strain. As can be seen, the addition of transcriptomics-based constraints to the model dramatically reduces the mean of the flux variability range, while the use of the metabolomics in the objective function did not cause any significant further reduction although it does cause the variability ranges to change slightly at some of the time points. Time points 41 and 97 had no significant metabolomics shifts thus the scenarios are the same with and without metabolomics. **(B)** We evaluated the use of constraints to include metabolomics information as compared to the use of the objective function to include the metabolomics information, by comparing the overall growth rate (biomass production) in four different conditions: Metab FBA (with metabolomics as objective function), Metab FBA with added constraints, Standard FBA (only transcriptomics information) and Standard FBA with constraints added from the metabolomics information but no objective function (similar to TREM-flux approach). It is clear that for some time points (65, 89, 137 and 257), the addition of the metabolomics-based constraints substantially reduces the ability of the model to create biomass.

Supplementary Tables are included as named sheets in the Supplementary Tables Excel file.

Supplementary Table 1: Fluxes obtained for FEM and GEM when applying standard FBA.

Supplementary Table 2: Fold enrichment of fluxes obtained using standard FBA and Metab FBA for FEM day 5 compared to day 10.

Supplementary Table 3: Fluxes obtained when using FBA IDH-1/2 KO in FEM when applying standard FBA.

Supplementary Table 4: Metabolomics data normalised

Supplementary Table 5: Results of PLS-DA on our metabolomics data showing 44 significantly changed metabolites with annotations with regards to their involvement in longevity. "SIG" indicates that the metabolite was found to change significantly with age, and those rows that are significant are also highlighted in grey. Of the significant rows, some are marked as INCREASING and others as DECREASING depending on the cluster they fell into.

Supplementary Table 6: Comparison of the metabolomics data with other similar studies. Grey highlighted rows are in disagreement with some published finding, while orange highlighted rows are in agreement with all published findings.

Supplementary Table 7: Fluxes through FEM and GEM when using metab_FBA.

Supplementary Table 8: Fluxes obtained when using FBA_metab_no_OAA.

Supplementary Table 9: Fluxes obtained when using FBA_metab_no_GLUT

Supplementary Table 10: Fluxes through TCA reactions using Standard FBA, Metab_FBA and Metab_FBA_ no_OAA.

Supplementary Table 11: Fluxes through production and consumption of gultamate (GLU) related to TCA cycle activity.

Supplementary Table 12: Metabolite names used in WormJam model.

Supplementary Table 13: Reactions names as used in WormJam model.

Supplementary Table 14: RNA Library Metrics.

Supplementary ZIP file: "Scripts, model and data files.zip" includes all the scripts, model and data files required to reproduce these results. The included files are (a) the model file (wormjam-20180125.sbml,input-reaction-bounds.csv), (b) the metabolomics differences in a CSV file (metabo-diffs.csv), (c) the transcriptomics data means (GroupMeans.csv), (d) the gene information used to map between the transcriptomics and the model (GeneInfoForModelGenes.csv,genes-

notfound.csv,metabo-model-mapping.csv), (e) Python script for running FBA (FBAPipeline.py), (f) R script and associated data files for processing the metabolomics data (ProcessMetabolomicsData.R,2017-09-06_(Mains_UK_65)_RelativeQuant.csv,all.sampleInfo.csv).

References

Anders, S., and Huber, W. (2010). Differential expression analysis for sequence count data. *Genome Biol.* doi:10.1186/gb-2010-11-10-r106.

Barardo, D., Thornton, D., Thoppil, H., Walsh, M., Sharifi, S., Ferreira, S., et al. (2017). The DrugAge database of aging-related drugs. *Aging Cell*. doi:10.1111/acel.12585.

Han, S., Schroeder, E. A., Silva-García, C. G., Hebestreit, K., Mair, W. B., and Brunet, A. (2017). Mono-unsaturated fatty acids link H3K4me3 modifiers to C. elegans lifespan. *Nature* 544, 185–190. doi:10.1038/nature21686.

Luo, W., and Brouwer, C. (2013). Pathview: an R/Bioconductor package for pathway-based data integration and visualization. *Bioinformatics* 29, 1830–1831. doi:10.1093/bioinformatics/btt285.

Luo, W., Friedman, M. S., Shedden, K., Hankenson, K. D., and Woolf, P. J. (2009). GAGE: generally applicable gene set enrichment for pathway analysis. *BMC Bioinformatics* 10, 161. doi:10.1186/1471-2105-10-161.

Schrier Vergano, S., Rao, M., McCormack, S., Ostrovsky, J., Clarke, C., Preston, J., et al. (2014). In vivo metabolic flux profiling with stable isotopes discriminates sites and quantifies effects of mitochondrial dysfunction in C. elegans. *Mol. Genet. Metab.* doi:10.1016/j.ymgme.2013.12.011.

Thévenot, E. A., Roux, A., Xu, Y., Ezan, E., and Junot, C. (2015). Analysis of the Human Adult Urinary Metabolome Variations with Age, Body Mass Index, and Gender by Implementing a Comprehensive Workflow for Univariate and OPLS Statistical Analyses. *J. Proteome Res.* doi:10.1021/acs.jproteome.5b00354.

Wold, S., Sjöström, M., and Eriksson, L. (2001). PLS-regression: A basic tool of chemometrics. in *Chemometrics and Intelligent Laboratory Systems* (Elsevier), 109–130. doi:10.1016/S0169-7439(01)00155-1.

Zhang, B., Kirov, S., and Snoddy, J. (2005). WebGestalt: An integrated system for exploring gene sets in various biological contexts. *Nucleic Acids Res.* 33, 741–748. doi:10.1093/nar/gki475.
